# Selective aggregation of PAMAM dendrimer nanocarriers and PAMAM/ZnPc nanodrugs on human atheromatous carotid tissues: a photodynamic therapy for atherosclerosis

**DOI:** 10.1186/s11671-015-0904-5

**Published:** 2015-05-07

**Authors:** Nikolaos Spyropoulos-Antonakakis, Evangelia Sarantopoulou, Panagiotis N Trohopoulos, Aikaterina L Stefi, Zoe Kollia, Vassilios E Gavriil, Athanasia Bourkoula, Panagiota S Petrou, Sotirios Kakabakos, Vadim V Semashko, Alexey S Nizamutdinov, Alkiviadis-Constantinos Cefalas

**Affiliations:** National Hellenic Research Foundation, Theoretical and Physical Chemistry Institute, 48 Vassileos Constantinou Avenue, Athens, GR-11635 Greece; CosmoPhosLtd, 77 Tsimiski Street, Thessaloniki, GR-54622 Greece; N.C.S.R. ‘Demokritos’, Institute for Nuclear and Radiological Sciences, Energy, Technology and Safety, Patriarchou Grigoriou Street, Athens, GR-15310 Greece; Institute of Physics, Kazan Federal University, 18 Kremljovskaja Street, Kazan, 420008 Russia

**Keywords:** Nanodrugs, Dendrimers, Nanoparticle aggregation, AFM, Fractal analysis, Biosurfaces, Atheromatous plaque, Photodynamic therapy, Cardiovascular, Surface roughness parameters

## Abstract

Photodynamic therapy (PDT) involves the action of photons on photosensitive molecules, where atomic oxygen or OH^−^ molecular species are locally released on pathogenic human cells, which are mainly carcinogenic, thus causing cell necrosis. The efficacy of PDT depends on the local nanothermodynamic conditions near the cell/nanodrug system that control both the level of intracellular translocation of nanoparticles in the pathogenic cell and their agglomeration on the cell membrane. Dendrimers are considered one of the most effective and promising drug carriers because of their relatively low toxicity and negligible activation of complementary reactions. Polyamidoamine (PAMAM) dendrite delivery of PDT agents has been investigated in the last few years for tumour selectivity, retention, pharmacokinetics and water solubility. Nevertheless, their use as drug carriers of photosensitizing molecules in PDT for cardiovascular disease, targeting the selective necrosis of macrophage cells responsible for atheromatous plaque growth, has never been investigated. Furthermore, the level of aggregation, translocation and nanodrug delivery efficacy of PAMAM dendrimers or PAMAM/zinc phthalocyanine (ZnPc) conjugates on human atheromatous tissue and endothelial cells is still unknown.

In this work, the aggregation of PAMAM zero generation dendrimers (G0) acting as drug delivery carriers, as well as conjugated G0 PAMAM dendrimers with a ZnPc photosensitizer, to symptomatic and asymptomatic human carotid tissues was investigated by using atomic force microscopy (AFM). For the evaluation of the texture characteristics of the AFM images, statistical surface morphological and fractal analytical methodologies and Minkowski functionals were used. All statistical quantities showed that the deposition of nanodrug carriers on healthy tissue has an inverse impact when comparing to the deposition on atheromatous tissue with different aggregation features between G0 and G0/ZnPc nanoparticles and with considerably larger G0/ZnPc aggregations on the atheromatous plaque. The results highlight the importance of using PAMAM dendrimer carriers as a novel and promising PDT platform for atherosclerosis therapies.

**Electronic supplementary material:**

The online version of this article (doi:10.1186/s11671-015-0904-5) contains supplementary material, which is available to authorized users.

## Background

Atherosclerosis is a common source of many cardiovascular diseases that cause stroke and heart attack. Atherosclerosis inflammation starts in the intima and media layers of the arterial wall section by forming a plaque, eventually leading to an abrupt rupture of the arterial wall. Although many pharmaceutical agents are available to treat different manifestations of atherosclerosis, their systemic delivery has serious disadvantages, including considerable side effects and low efficacy at tolerated doses [1]. Recent advances in nanotechnology have provided new tools for the efficient diagnosis and therapy of atherosclerosis [2-7]. Photodynamic therapy (PDT), which uses functionalized nanoparticles (NPs) that are selectively attached to the diseased tissues or cells, is a promising method for the localized treatment of atherosclerosis [8].

The action of PDT consists of the selective attachment of photosensitizing molecules (PSs) to the diseased tissue. Irradiation of tissues at specific wavelengths of light activates the PSs, thus promoting cell death [9]. Once irradiated, a PS molecule is transferred into an excited molecular electronic state by the absorption of one photon and eventually transfers its energy to its surrounding molecular oxygen, generating reactive oxygen species (ROS), either singlet oxygen or OH^−^ molecules [10,11]. Due to its short life-time, ROS diffuse within the tissue at a distance of up to few tenths of a nanometer; therefore, the biological damage is limited to the sub cellular location of the action of PSs [12].

**Figure 1:**
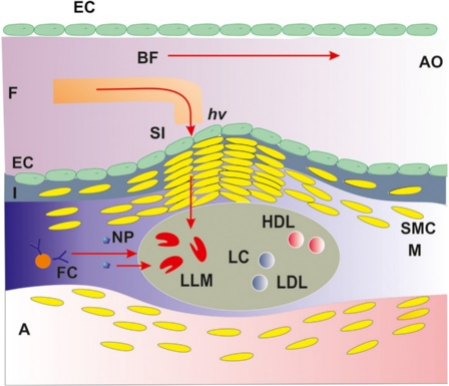
Simplified schematic lay-out of PDT in atherosclerosis. The lipid core plaque (LC) is responsible for stenosis of the aorta (AO). Photons (*hv*) guided by a fibber (F) illuminate the microphage cells in the plaque (LLM), inducing necrosis following nanoparticle (NP) uptake. SI, side illumination of atheromatous plaque; I, intima; SMC, smooth muscle cells; M, media; A, adventida; FC, foam cell; BF, blood flow; EC, endothelial cells; LDL, low-density lipoprotein; HDL, high-density lipoprotein.

The rationale for using PDT in atherosclerosis is justified by a few experimental results [13], where different PSs, such as porphyrins and phthalocyanines (Pcs), are shown to selectively accumulate in the atherosclerotic plaque rather than in the adjacent normal vessel wall (e.g. endothelium), as seen in Figure 1. The overall aim of PDTs is to stop the biological activities of macrophage cells in atheromatous plaques by necrosis, as described previously in the work of McCarthy et al. [14-17] (Figure 1).

Furthermore, various PS phthalocyanine molecules have advantageous photophysical properties [18], owing to the selectivity of light absorption at 670 nm, where the depth of photon penetration in tissues is twice as high as the penetration depth of porfimer sodium (Photofrin) at 630 nm [19]. In PDTs, the singlet oxygen’s quantum yield is high enough [20] to account for a considerable cell necrosis.

Despite the fact that most of the PSs used in PDTs have low biocompatibility and water-solubility, which limit their effective clinical usage [21,22], both issues are addressed via conjugation of PSs with proper nanocarrier materials [23-25]. The main advantages of using polymer-PS nanodrug conjugates are the high water solubility of the hydrophobic drugs, controlled drug release under certain conditions (e.g. pH or presence of enzymes), resistivity of drugs against degradation, prolonged plasma life-time, improved biocompatibility, altered biodistribution and either specific accumulation in localized sites or enhanced permeability and retention (EPR) in cells [26]. However, in any conjugated system, strong aggregation or self-association of the PSs occurs, leading to detrimental effects, such as low drug delivery efficacy or even tissue damage [27,28].

In the special case of zinc phthalocyanine (ZnPc), successful conjugation with different nanocarriers has already been demonstrated in many other studies [25,29-36], and dendritic delivery of PDT agents has been investigated within the last few years to improve upon tumour selectivity, retention and pharmacokinetics [37,38].

Because of their unique synthetic roots, dendrimers combine a defined composition and monodispersity with a high molecular mass, giving them interesting physical and chemical properties [39]. A large number of functional end-groups are responsible for high solubility and reactivity. Internal cavitations at the nano/microscale can be utilized in transporting small molecules as well [40-42].

Polyamidoamine (PAMAM) molecules comprise a novel class of spherical, biocompatible, safe, non-immunogenic and highly branched or cascade polymers [42] studied as model nanoparticles in biomedical applications, including drug delivery [43-45], owing to their relative large nanometric size and water solubility [46]. Moreover, PAMAM dendrimers can induce multivalence effects, similar to the polyvalent interactions in biological systems [47], but any successful PDT methodology in atherosclerosis must avoid drug aggregation on the endothelial cells (Figure 1). In addition, the efficacy of penetration into the endothelial cells, as well as the selectivity, aggregation and translocation of PAMAM dendrimers or PAMAM/ZnPc conjugates, on human atheromatous tissue is unknown. Recently, it was established that the efficiency of intracellular nanodrug uptake in a cell and the level of agglomeration on the cell membrane are functions of the local nanothermodynamic conditions near the cell-wall/nanodrug system [28].

In this work, the adhesion and aggregation characteristics of different zero generation PAMAM dendrimers (G0) acting as drug delivery carriers, as well as conjugated G0 PAMAM dendrimers with a ZnPc photosensitizer (G0/ZnPc), to symptomatic (atheromatous) and asymptomatic (healthy) human carotid tissues were studied by using atomic force microscopy (AFM), statistical surface analysis and different fractal analytical methodologies. For the evaluation of the texture characteristics of the AFM images and the adhesion and aggregation characteristics of the nanodrugs, scaling from 1 × 1 μm to 5 *×* 5 μm, fractal analytical methodologies (variance, power spectrum, cube counting and triangulation) and Minkowski functionals (volume, boundary, connectivity) were used, together with statistical surface analysis, allowing for differentiation in the selective aggregation of nanodrugs in symptomatic and asymptomatic carotid tissues.

## Methods

### G0 PAMAM dendrimers

A G0 PAMAM dendrimer (1,4-diaminobutane core, carbomethoxy pyrolidinone terminated, GEN 0.0, C_48_H_76_N_10_O_16_) (NanoSynthons, LLC, Mt Pleasant, Michigan, USA) in lyophilized form was used. A quantity of 60 mg was resolved in 1 ml of distilled water, resulting in a concentration of 0.05 M. Then, the solution was further diluted to a final concentration of 0.025 M.

### Tissues

Carotid tissues were kindly offered by Poznan University of Medical Sciences, Poland. They were surgically removed from patients who had atheromatous plaques near the common carotid artery and also from patients who had an atheromatous plaque in other areas of the artery; the major carotid artery branch was asymptomatic. Tissues stained in paraformaldehyde were shipped and preserved in 70% ethanol. They were hydrated with Ringer’s solution with the osmotic pressure as blood (mixture of NaCl, KCl, CaCl_2_ and NaHCO_3_), and then a microsection of 60 μm was acquired both in the asymptomatic and symptomatic area of the carotid. The microsection was placed on a silica wafer or glass and was poured for 30 min with the aquatic solution of G0 dendrimers or conjugated molecules of ZnPc and G0 dendrimers. Samples were treated according to the ethic issues specified in the FP7-NMP-2012-LARGE-6 ‘CosmoPhos-Nano’ project (reference number: 310337). The study was approved by the ethics committee of Poznan University of Medical Sciences, Poland.

### Photonic conjugation of G0 dendrimer and ZnPc

**Figure 2:**
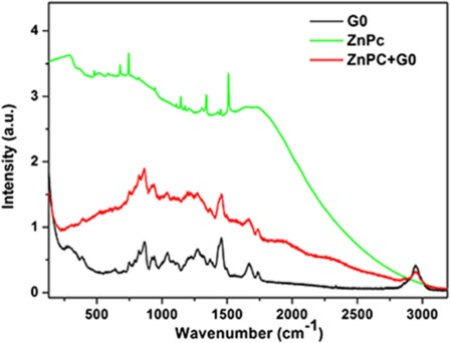
μ-Raman spectra (100 to 3,200 cm^−1^, 850-nm diode laser) G0 and ZnPc solution. The Raman spectra of G0 and ZnPc powder are also shown for comparison. The new bands around 1,923 cm^−1^ and 2,298 cm^−1^ can be attributed to the photo-conjugated G0/ZnPc.

Zinc phthalocyanine (Sigma-Aldrich, St. Louis, MO, USA) was used as the active part of the nanodrug. Photonic conjugation of the dendrimer and ZnPc was achieved with laser light at 157 nm (LPF 200, Lambda-Physik (since 2006), Coherent, Santa Clara, CA, USA) [48,49] to avoid the use of additional catalysts that might enhance agglomeration; ZnPc/G0 dendrimer solutions were prepared by diluting 4 × 10^−5^ kg of lyophilized G0 in 10^−3^ l H_2_O in a final concentration of 0.038 M. Subsequently, ZnPc (MW = 577.91 × 10^−3^ kg) was partially diluted in 5 × 10^−3^ l phosphate-buffered saline (PBS) in a final concentration of 0.034 M. The PBS buffer was selected for its biocompatibility to avoid toxicity. Two parts of the G0 solution were mixed with one part ZnPc. To avoid the creation and consequent precipitation of aggregated G0 and ZnPc, the solution was sonicated for 30 min. A total of 1.5 × 10^−3^ l of the final solution was placed into a cell that had CaF_2_ windows, which are transparent at VUV wavelengths. The cell was installed in front of the 157-nm (7.8 eV) laser. The energy, fluence, pulse duration at full width at half maximum and the repetition rate of the unfocused laser beam per laser pulse were 24 mJ, 75 Jm^−2^, 15 ns and 15 Hz, respectively. At 157 nm, there is a complete bond breaking of all the organic molecules [50]. The parent molecule disintegrates into small fragments that are atomic, diatomic or triatomic. This is because the dissociative excited states of the small radicals occupy the energy range above 6.5 eV (200 nm). This unique functionality at low wavelengths and 157 nm allows for the unique bio-functionalities of surfaces [51,52]. Following laser irradiation at 157 nm, a drop of the liquid was deposited on a Si wafer and left to dry. Then, the film was studied by μ-Raman spectroscopy (100 to 3,200 cm^−1^, 850-nm diode laser). The μ-Raman spectra of G0 and ZnPc powder are also included in Figure 2 for comparison. The new bands at approximately 1,923 cm^−1^ and 2,298 cm^−1^ were attributed to photoconjugated G0/ZnPc as a result of the formation of new bonds following the dissociation of the parent molecules.

### Atomic force microscopy

**Figure 3:**
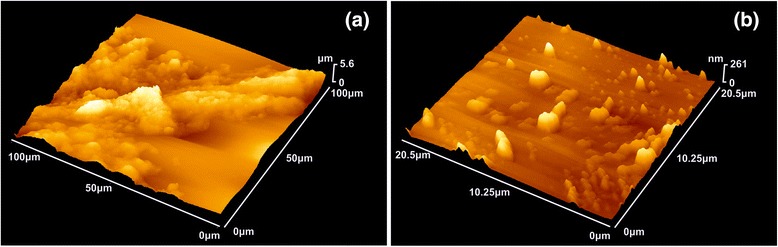
3D-AFM image of atheromatous plaque. **(a)** 100 × 100 μm size image. **(b)** 20.5 × 20.5 μm size image, higher magnification of Figure 3a.

Images of symptomatic and asymptomatic carotids, as well as symptomatic and asymptomatic carotids with the addition of the G0 and G0/ZnPc conjugates were acquired under ambient conditions by using an AFM (d’Innova, Bruker, Madison, WI, USA). AFM provides high-resolution imaging and measurements of surface topography and properties at the nanoscale. The AFM images of symptomatic/asymptomatic carotids were acquired in tapping-mode using a phosphorus-(n)-doped silicon cantilever (RTESPA, Bruker, Madison, WI, USA) with a nominal spring constant of 40 N/m at approximately 300 kHz resonance frequency and a nominal radius of 8 nm (Figure 3). The AFM images were obtained at different scanning areas at a maximum scanning rate of 0.5 Hz with an image resolution of 512 × 512 pixels. Imaging was carried out at different scales from 1 to 5 μm to verify the consistency and robustness of the evaluated structures. The AFM data were processed with WSXM™ free software [53].

### Surface roughness parameters

The root mean square surface roughness (*R*_*q*_), the surface roughness (*R*_*a*_), the mean $\left(\overline{Z}\right),$ media (*Z*_1/2_), mode (*Z*_*mp*_) and range (*R*_*t*_) heights, the maximum valley depth (*R*_*mvd*_), the maximum peak height (*R*_*mph*_), skewness (*R*_*sk*_) and kurtosis (*R*_*ku*_) are used to quantify surface roughness. The statistical parameters of surfaces are shown in Additional file 1 and partially in Table 1. They are calculated from the equations ${R}_q=\sqrt{\frac{1}{N}{\displaystyle \sum }{\left({Z}_i-\overline{Z}\right)}^2},\cdots {R}_a=\frac{1}{N}{\displaystyle \sum}\left|{Z}_i-\overline{Z}\right|,\kern0.5em \overline{Z}=\frac{1}{N}{\displaystyle \sum}\left|{Z}_i\right|$. The *Z*_1/2_ and *Z*_*mp*_ parameters are calculated from the probability distribution function (PDF) of heights *f*(*Z*) that satisfy the equations ${\int}_{-\infty}^{Z_{1/2}}f(Z)dZ={\int}_{Z_{1/2}}^{\infty }f(Z)dZ=1/2$ and $\frac{d}{dZ}{\int}_{-\infty}^{\infty }f(Z)dZ=0$.

**Table 1:** **Surface characteristics obtained from AFM images**

	$\overline{\boldsymbol{Z}}\left(\boldsymbol{nm}\right)$	***R***_***a***_ **(** ***nm*** **)**	***R***_***q***_ **(** ***nm*** **)**
1 × 1 μm			
Healthy	24.91	7.78	9.96
Atheromatous	49.37	7.87	10.12
Healthy + PAMAM	8.74	1.62	2.18
Glass + PAMAM (0.05 M)	3.54	0.53	0.79
Glass + PAMAM (0.025 M)	1.89	0.20	0.38
2 × 2 μm			
Healthy	78.92	15.77	20.12
Atheromatous	56.46	12.90	16.41
Healthy + PAMAM	41.49	3.65	5.48
Atheromatous + PAMAM	166.63	17.23	24.09
Atheromatous + PAMAM + ZnPc	175.17	32.37	41.97
3 × 3 μm			
Atheromatous	102.26	16.96	21.22
Atheromatous + PAMAM + ZnPc	280.84	46.62	60.30
5 × 5 μm			
Healthy	133.98	34.58	43.20
Atheromatous	166.30	30.81	39.06
Atheromatous + PAMAM	266.32	50.96	65.86
Atheromatous + PAMAM + ZnPc	260.82	69.35	87.03
Glass + PAMAM (0.025 M)	19.23	2.98	6.57

AFM, atomic force microscopy; PAMAM, polyamidoamine; ZnPc, zinc phthalocyanine.

Additionally, *R*_*t*_ = |*Z*_*max*_ − *Z*_*min*_|, ${R}_{mvd}=\left|{\left({Z}_i-\overline{Z}\right)}_{min}\right|,\kern0.5em {R}_{mph}={\left({Z}_i-\overline{Z}\right)}_{max}, \kern0.24em {R}_{sk}=\frac{1}{N{R}_q^3}{\displaystyle \sum_{i=0}^N}{\left|{Z}_i-\overline{Z}\right|}^3,\kern0.5em {R}_{ku}=\frac{1}{N{R}_q^4}{\displaystyle \sum_{i=0}^N}{\left|{Z}_i-\overline{Z}\right|}^4$, where *Z*_*i*_ is the *i*th height and *N* is the number of data points over which *Z*_*i*_ is collected.

Recently, the surface roughness of arteries acquired from ultrasound images was used for the early diagnosis of atherosclerosis [54]. However, it can only be used for qualitative investigations because they differ by the scale of measurement. It also does not include information about the spatial distribution of the topography.

### Fractal methods

The use of fractal geometry, with concepts such as self-similarity and self-affinity, introduced by Mandelbrot [55], has changed and broadened the characterization of surfaces as well as many phenomena and processes. Today, several algorithms have been developed in the literature for the estimation of fractal dimensions from the AFM images. The fractal dimension *D*_*f*_ is a measure of the degree of variation of a surface from its topological ideal and is related to both surface roughness and its topological entropy. It describes the degree of chaoticity of a surface and is a number lying between 2 for a smooth surface and 3 for an extremely rough surface [56].

There are many applications of fractal analysis for the characterization of biological surfaces or structures using magnetic resonance imaging [57-59], ultrasound [60,61] or other techniques [62,63]. However, evaluations of fractal parameters from AFM images for biological surfaces are scarce [64,65]. For example, Bitler et al. [64] evaluated the fractal dimension of the macrophage cell membrane and tested the sensitivity of the fractal dimension value to submicron changes in membrane morphology using high-resolution AFM imaging.

In this work, the fractal dimension *D*_*f*_ was calculated from the AFM data using B-Spline interpolation with four methods, i.e. cube counting, triangulation, variance and power spectrum distribution, using the free and open source software program Gwyddion [66]. For the four methods, all of the slopes were determined using a linear fit. Before fractal analysis, all AFM images were processed using a dilation algorithm to compensate for the convolution between the tip and the surface and tip distortions caused by the orientation of the tip and the forces between the tip and surface [67]. For dilation, a contact tip with a pyramid shape and a tip apex of 12 nm that was rotated by 45° was used as the model tip for all surface scan reconstructions [68]. For fractal dimension estimation based on structure function and variation methods, we developed custom MATLAB (MathWorks Inc., Natick, MA, USA) codes. The original images were opened, and processed, and the fractal dimension was evaluated with these programs.

### Minkowski functionals

The Minkowski functionals were used to describe the global geometric characteristics of structures. Two-dimensional discrete variants of volume *V*, surface *S* and connectivity (Euler-Poincaré Characteristic) *x* were calculated using the Gwyddion™ software [66]. The Minkowski functionals provide parameters that allow for the fundamental quantification of the dispersion. They represent a method to describe the amount of connectivity by analysing the relationship of connected pixels and unconnected pixels present in an image. Minkowski functionals are very effective at describing complex morphologies observed in images [69-71].

## Results

### AFM morphology, RMS and height distribution

**Figure 4:**
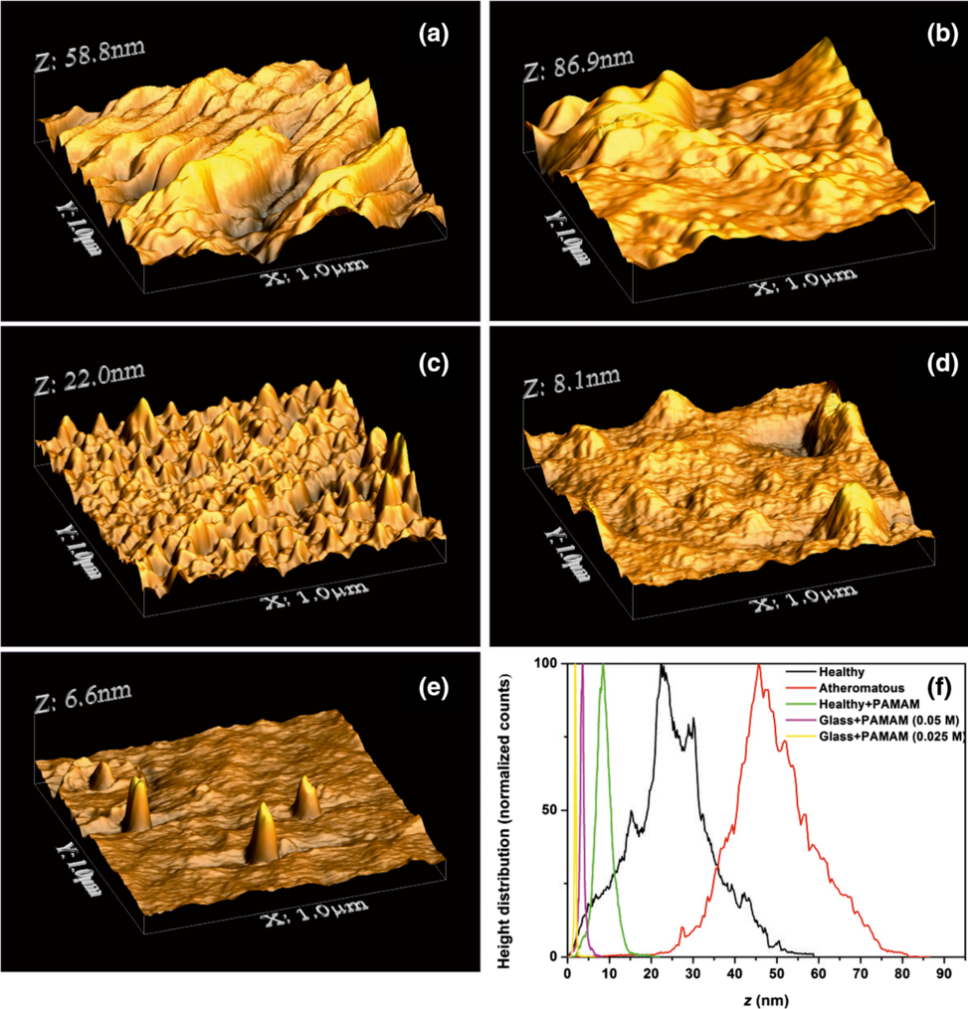
AFM images (1 × 1 μm) showing **(a)** a healthy carotid tissue, **(b)** an atheromatous carotid tissue, **(c)** a healthy carotid tissue with G0 PAMAM dendrimers, **(d)** a solution (0.05 M) of dendrimers on glass and **(e)** a highly diluted solution (0.025 M) of dendrimers on glass. **(f)** Height distribution of the **(a)**-**(e)** samples.

**Figure 5:**
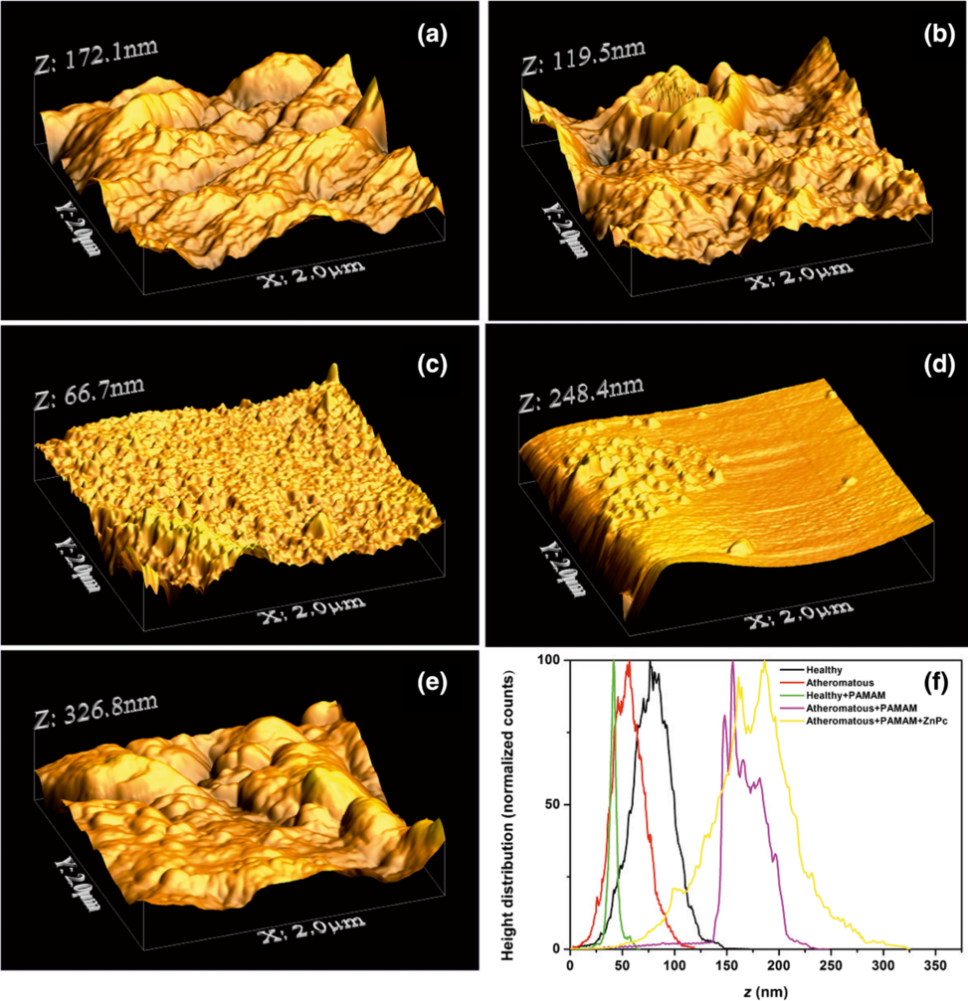
AFM images (2 × 2 μm) showing **(a)** a healthy carotid tissue, **(b)** an atheromatous carotid tissue, **(c)** a healthy carotid tissue with G0 PAMAM dendrimers, **(d)** an atheromatous carotid tissue with G0 PAMAM dedrimers and **(e)** an atheromatous carotid tissue with dedrimers conjugated with ZnPc. **(f)** The corresponding height distribution of the 2 × 2 μm samples.

**Figure 6:**
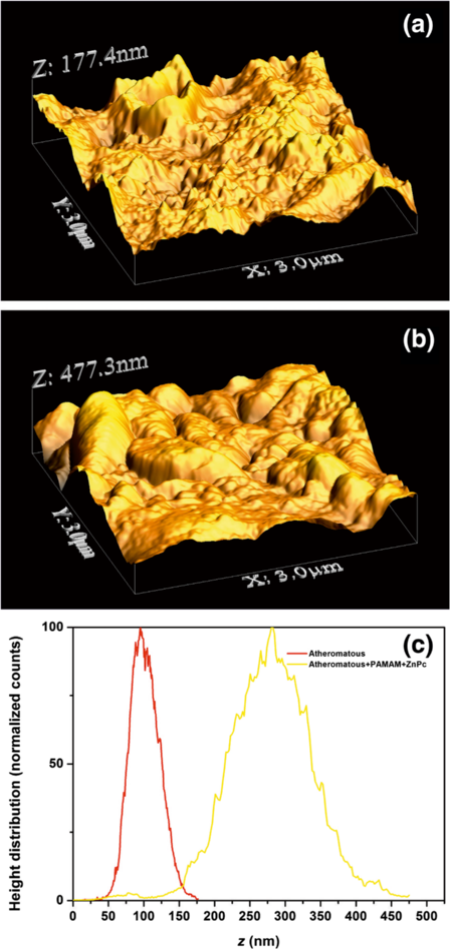
AFM images (3 × 3 μm) showing **(a)** an atheromatous carotid tissue, **(b)** an atheromatous carotid tissue with G0 PAMAM dedrimers conjugated with ZnPc. **(c)** The corresponding height distribution of the 3 × 3 μm samples.

**Figure 7:**
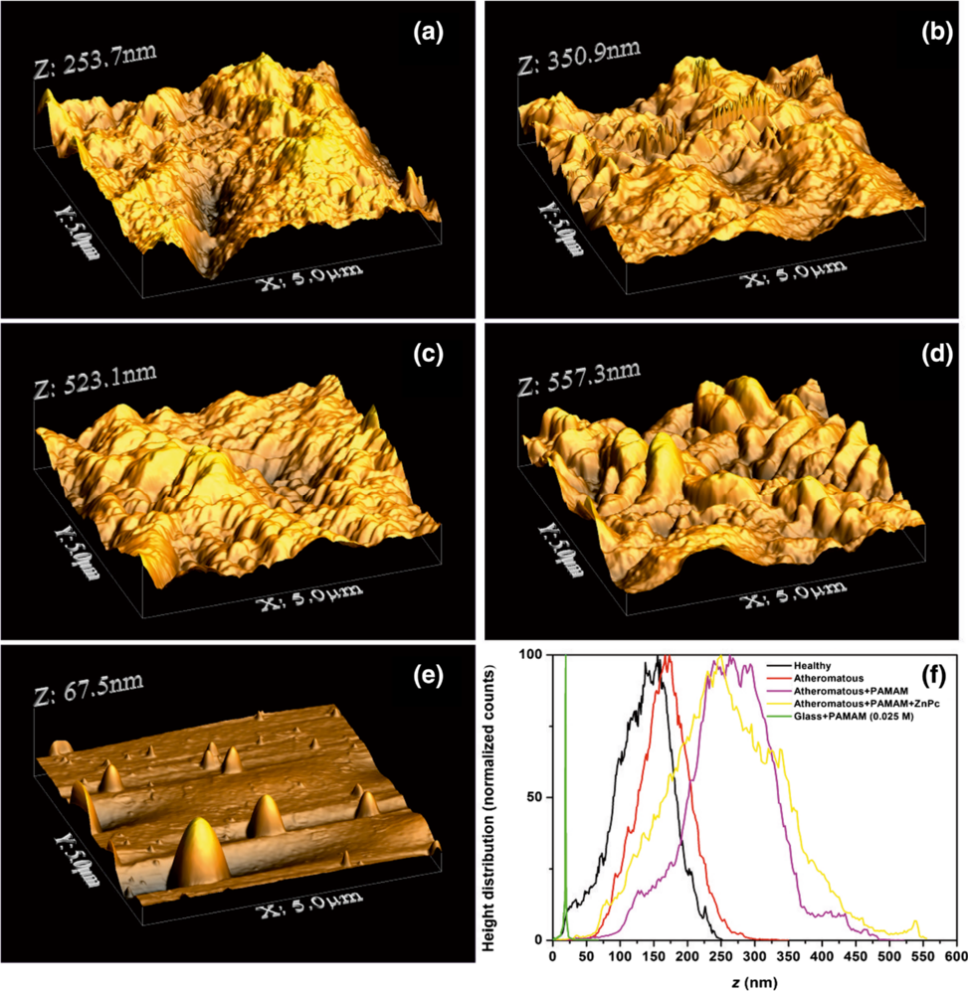
AFM images (5 × 5 μm) showing **(a)** a healthy carotid tissue, **(b)** an atheromatous carotid tissue, **(c)** an atheromatous carotid tissue with G0 PAMAM dedrimers, **(d)** an atheromatous carotid tissue with G0 PAMAM dedrimers conjugated with ZnPc and **(e)** a highly diluted solution (0.025 M) of dendrimers on glass. **(f)** The corresponding height distribution of the 5 × 5 μm samples.

It is found from Table 1, Figures 4, 5, 6 and 7 and Additional file 1 that the PDF of heights *f*(*Z*) is unimodal, because the statistical mean, media and mode values of height are almost the same. Plain 1 × 1 μm AFM images of a carotid tissue, either healthy (H), Figure 4a, or atheromatous (A), Figure 4b, display higher $\overline{Z}$ and *R*_*q*_ values than healthy carotid samples with G0 dendrimers (G0-H) (Figure 4c and Table 1). The corresponding $\overline{Z}$ values are approximately 8.74 nm (G0-H) versus approximately 24.91 nm (H) or approximately 49.37 nm (A). The *R*_*q*_ values are approximately 2.18 nm (G0-H) versus approximately 9.96 nm (H) or approximately 10.12 nm (A).

Deposition of the G0 dendrimer on a glass substrate, Figure 4d,e, indicates even lower roughness values, $\overline{Z}$ ~3.54 nm and *R*_*q*_~0.79 nm and $\overline{Z}$ ~1.89 nm and *R*_*q*_~0.38 *nm* for 0.05 and 0.025 M G0 concentrations, respectively. The cluster size distribution shifts to the right at higher concentrations (Figure 4f).

A similar trend of surface roughness values appears for the 2 × 2 μm AFM images (Figure 5a,b,c). For the G0 dendrimers on healthy and atheromatous tissues, $\overline{Z}$ ~41.49 nm (G0-H) and *R*_*q*_~24.09 nm (G0-H) versus $\overline{Z}$ ~78.92 nm (H) and *R*_*q*_~20.12 nm (H). In contrast, the deposition of G0 dendrimers on atheromatous carotid tissues, Figure 5d, results in higher surface roughness values, $\overline{Z}$ ~166.63 nm (G0-A) and *R*_*q*_~24.09 nm (G0-A), compared to the values of $\overline{Z}$ ~56.46 nm (A) and *R*_*q*_~16.41 nm (A).

The addition of the conjugated G0/ZnPc drug on the atheromatous carotid tissue, Figure 5e, has a similar impact on the surface roughness parameters as the addition of the plain G0 on atheromatous carotid tissue, although the *R*_*q*_ value is much higher, *R*_*q*_~41.49 nm. This tendency is strengthened by the results obtained from the 3 × 3 μm AFM images, where the $\overline{Z}$ and the *R*_*q*_ surface roughness values for the plain atheromatous carotid tissue, Figure 6a, are $\overline{Z}$ ~102.26 nm (A) and *R*_*q*_~21.2 nm (A). These surface roughness values are lower than those of the atheromatous tissue with the addition of the conjugated G0/ZnPc solutions, Figure 6b, where $\overline{Z}$ ~280.84 nm (G0/ZnPc) and *R*_*q*_~60.30 nm (G0/ZnPc). Finally, the 5 × 5 μm AFM images, Figure 7, also demonstrated that the addition of the G0 dendrimers, Figure 7c, or G0/ZnPc, Figure 7d, on the atheromatous tissue affects the surface roughness values significantly, indicating a higher level of agglomeration. On the contrary, the surface roughness values of the G0 NPs on the healthy tissue indicate a low level of agglomeration with average surface parameters that are smaller or equal to those of the healthy tissues. Overall, the results point out that the type of biosurface mediates the agglomeration efficiency of nanodrugs, in agreement with previous results [28].

Size statistics of aggregation of ZnPc nanoparticles on gold (Au) and Si standard test surfaces is different than the aggregation of G0 on the same surfaces. ZnPc nanoparticles tend to aggregate at average sizes larger than the surface roughness parameters of the test surfaces (Additional file 2). This response is owing to the presence of electron charges on the surface of ZnPc nanoparticles and demonstrated by conductive atomic force microscopy (Additional file 2). In case of conjugation of ZnPc with the G0 dendrimers, the negative charges are compensated to give nearly neutral nanoparticles of smaller size.

### Fractal analysis

**Figure 8:**
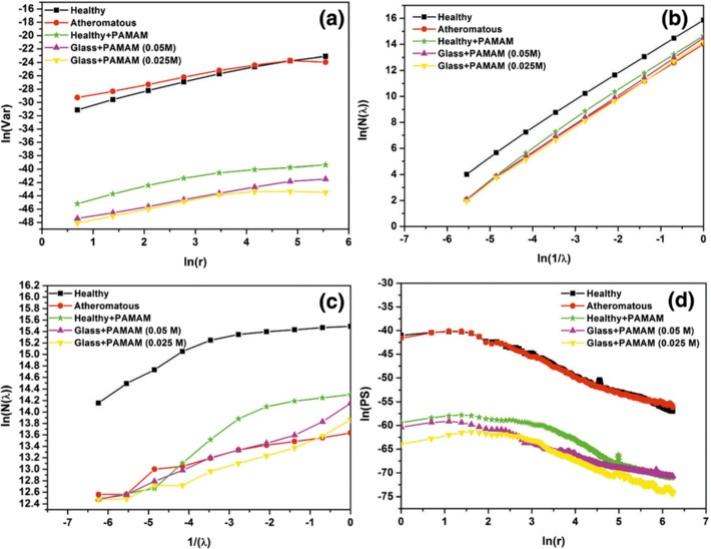
Log-log plots illustrating calculation of fractal dimension by different methods for the 1 × 1 μm AFM images. **(a)** Variance, **(b)** cube counting, **(c)** triangulation and **(d)** power spectrum.

**Figure 9:**
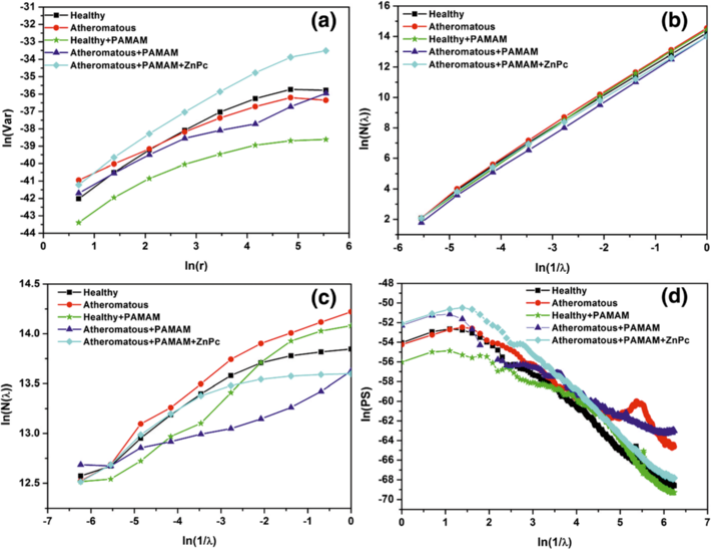
Log-log plots illustrating calculation of fractal dimension by different methods for the 2 × 2 μm AFM images. **(a)** Variance, **(b)** cube counting, **(c)** triangulation and **(d)** power spectrum.

**Figure 10:**
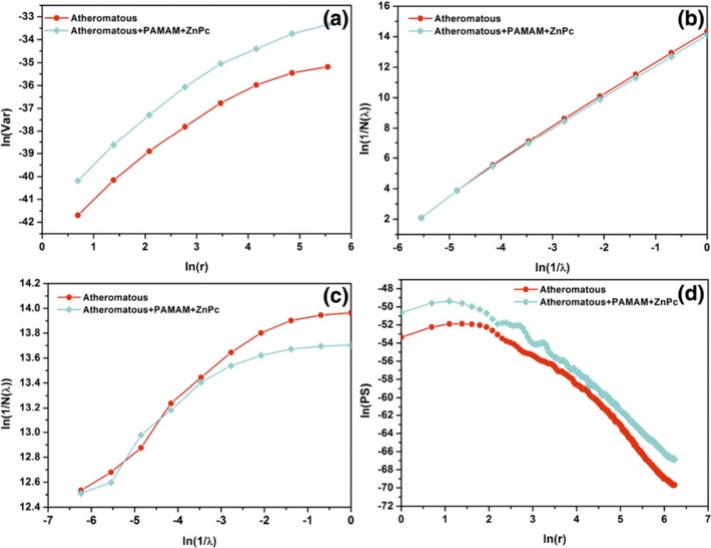
Log-log plots illustrating calculation of fractal dimension by different methods for the 3 × 3 μm AFM. **(a)** Variance, **(b)** cube counting, **(c)** triangulation and **(d)** power spectrum.

**Figure 11:**
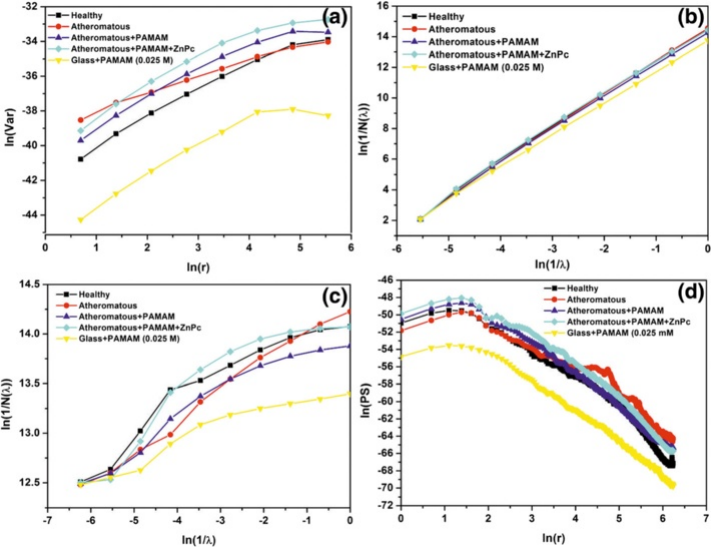
Log-log plots illustrating calculation of fractal dimension by different methods for the 5 × 5 μm AFM images. **(a)** Variance, **(b)** cube counting, **(c)** triangulation and **(d)** power spectrum.

In Figures 8, 9, 10 and 11, the log-log plots used for the calculation of fractal dimensions by applying different methodologies for the 1 × 1 μm, 2 × 2 μm, 3 × 3 μm and 5 × 5 μm sets of AFM images are illustrated. The evaluated fractal dimensions from the four different methods are presented in Table 2. In general, the three methods, namely, variance, cube counting and triangulation, give fractal dimensions that have a similar trend in most cases. In contrast, the power spectrum results not only differ from the other methods but also, most importantly, have values below 2; hence, this method is inappropriate to characterize the samples. It is most important that despite a difference in the absolute values calculated by the three different approaches, the effect of the deposition of G0 dendrimers is consistent. On all scales, a comparison between the images of healthy and atheromatous carotid samples shows that the healthy tissue has a lower fractal dimension than atheromatous, in agreement with Asvestas et al. [60], Rakebrandt et al. [61] and Niu et al. [54], who used statistical and textural measures based on fractal geometry to quantify texture features from ultrasound (US) B-mode images of carotid atherosclerotic plaques.

**Table 2:** **Fractal dimensions (** ***D***_***f***_ **) calculated by four different methods**

	**Variance**	**Cube counting**	**Triangulation**	**Power spectrum**
1 × 1 μm				
Healthy	2.23	2.18	2.23	1.68
Atheromatous	2.41	2.25	2.34	1.89
Healthy + PAMAM	2.42	2.13	2.18	2.01
Glass + PAMAM (0.05 M)	2.36	2.21	2.26	2.44
Glass + PAMAM (0.025 M)	2.49	2.19	2.22	2.05
2 × 2 μm				
Healthy	2.30	2.14	2.21	1.95
Atheromatous	2.52	2.24	2.29	1.66
Healthy + PAMAM	2.49	2.22	2.28	2.40
Atheromatous + PAMAM	2.44	2.17	2.14	2.43
Atheromatous + PAMAM + ZnPc	2.19	2.13	2.18	1.56
3 × 3 μm				
Atheromatous	2.32	2.19	2.25	1.29
Atheromatous + PAMAM + ZnPc	2.30	2.14	2.20	1.50
5 × 5 μm				
Healthy	2.28	2.22	2.26	1.47
Atheromatous	2.53	2.23	2.30	1.84
Atheromatous + PAMAM	2.33	2.18	2.24	1.64
Atheromatous + PAMAM + ZnPc	2.33	2.19	2.28	1.47
Glass + PAMAM (0.025 M)	2.34	2.08	2.14	1.73

PAMAM, polyamidoamine; ZnPc, zinc phthalocyanine.

Furthermore, when G0 dendrimers are deposited onto healthy tissue, the fractal dimension is increased, whereas when they are deposited onto atheromatous carotid tissues, the fractal dimension is reduced.

Moreover, when conjugated G0 dendrimers with ZnPc are deposited onto athermanous carotid tissues, the fractal dimension is reduced further.

Finally, images of the G0 dendrimers on symptomatic or asymptomatic tissue give higher fractal dimensions than images of the G0 dendrimers (0.05 M or 0.025 M) deposited onto plain glass, demonstrating that both substrate type and roughness greatly mediate the agglomerating behaviour of the drugs.

### Minkowski functionals

**Figure 12:**
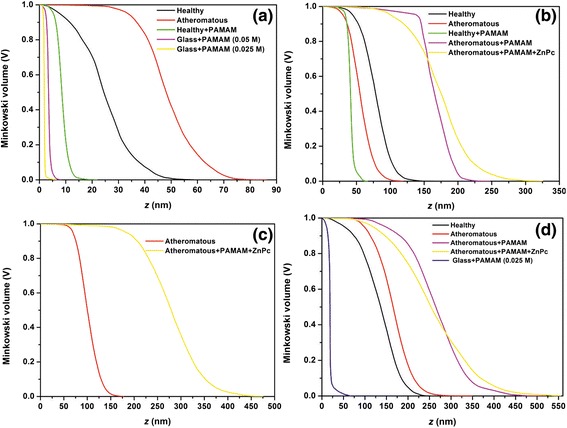
Minkowski volume of **(a)** 1 × 1 μm, **(b)** 2 × 2 μm, **(c)** 3 × 3 μm and **(d)** 5 × 5 μm AFM images.

**Figure 13:**
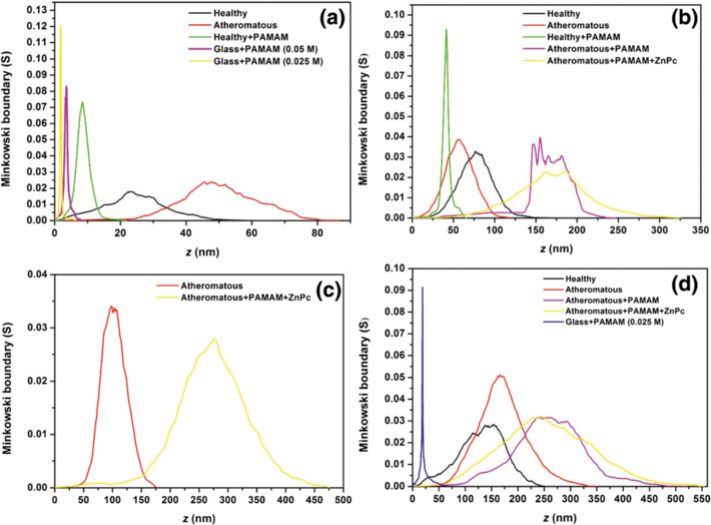
Minkowski boundary of **(a)** 1 × 1 μm, **(b)** 2 × 2 μm, **(c)** 3 × 3 μm and **(d)** 5 × 5 μm AFM images.

**Figure 14:**
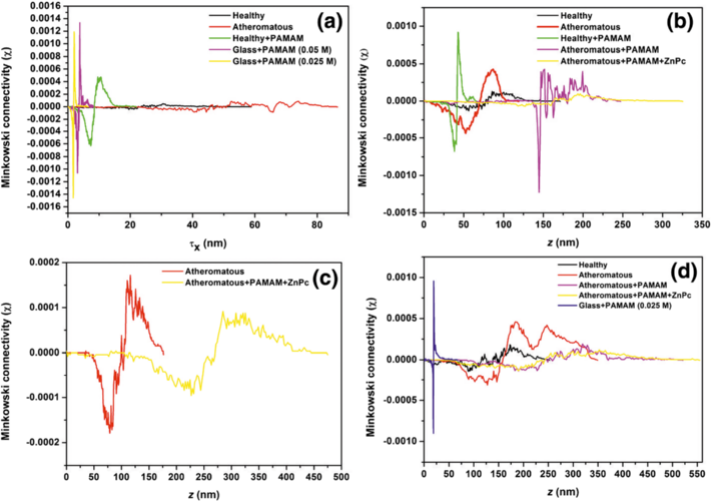
Minkowski connectivity of **(a)** 1 × 1 μm, **(b)** 2 × 2 μm, **(c)** 3 × 3 μm and **(d)** 5 × 5 μm AFM images.

The Minkowski functionals, i.e. the volume *V*, Figure 12, the boundary length *S*, Figure 13 and the connectivity (Euler characteristic) *x*, Figure 14, are shown for the AFM images of Figures 4, 5, 6 and 7. The Minkowski volume curves in Figure 12 represent the volume of the rough regions, i.e. the number of pixels with a height threshold larger than *z*, and they are decreased monotonously from 1 to 0. For the set of 1 × 1 μm AFM images, Figure 12a, it is shown that, in contrast to the carotid tissue that can be either healthy or atheromatous, which both show a nearly point symmetric behaviour with respect to *V* = 0.5, *Z* = 48 nm and *Z* = 24 nm, respectively, the samples that contained the deposited PAMAM dendrimers favour the black areas. For the set of 2 × 2 μm AFM images, Figure 12b, it is shown that symptomatic and asymptomatic carotid tissues, as well as atheromatous carotid tissue with PAMAM dendrimers conjugated with ZnPcs, show a nearly point symmetric behaviour with respect to *V* = 0.5 and *Z* = 55 nm, *Z* = 79 nm and *Z* = 176 nm, respectively. The carotid tissues where only G0 dendrimers were deposited are shown to have an asymmetrical behaviour. More specifically, the healthy tissue with dendrimers favours the black areas (smooth regions), while the atheromatous one favours white areas (rough regions). For the set of 3 × 3 μm AFM images, Figure 12c, it is shown that atheromatous carotid tissue and atheromatous carotid tissue with G0 dendrimers conjugated with ZnPcs show a nearly point symmetric behaviour with respect to *V* = 0.5 and *Z* = 100 nm and *Z* = 278 nm, respectively. The same trend is observed in the curves of Figure 12d for the 5 × 5 μm AFM images. The curves of the plain (healthy or atheromatous) or G0/ZnPc-treated tissue show a nearly point symmetric behaviour with respect to *V* = 0.5 and *Z* = 165 nm, *Z* = 136 nm and *Z* = 253 nm, respectively, while the samples (tissue or glass) where only dendrimers were deposited show an asymmetrical behaviour.

The Minkowski boundary length, Figure 13, tends to zero for nearly flat and most rough areas and reaches its maximum in the intermediate threshold range. For the set of 1 × 1 μm AFM images, Figure 13a, it is shown that the samples containing deposited G0 dendrimers have narrow and intense Gaussian curves, while the carotid tissue that is either symptomatic or asymptomatic shows asymmetric broad and weak peaks. For the set of 2 × 2 μm AFM images, Figure 13b, it is shown that healthy and atheromatous tissue have Gaussian curves, healthy carotid tissue with G0 dendrimers has a narrow and more intense Gaussian peak and the deposition of G0 dendrimers on atheromatous carotid tissue results in a very asymmetric peak. However, the addition of the ZnPc nanodrug tends to give a nearly symmetrical broad curve with two peaks (non-Gaussian). For the set of 3 × 3 μm AFM images, Figure 13c, it is shown again that the deposition of G0 conjugated with ZnPc tends to give more broad curves when compared to the plain atheromatous carotic. The same trend is also verified in the set of 5 × 5 μm AFM images, Figure 13d.

The Minkowski connectivity (Euler characteristic *x*) describes the topological structure of the pattern. It is negative (positive) if many disconnected flat (rough) components dominate the image. A vanishing Euler characteristic indicates a highly connected structure with an equal amount of flat and rough components [70]. In Figure 14a (1 × 1 μm), the Minkowski connectivity decreases very sharply at *τ*_*χ*_ = 1.4 (2.0) to the minimum negative value at *τ*_*χ*_ = 1.7 (3.1) nm for the images of G0 dendrimers on glass with a concentration of 0.025 M (0.05 M). Then, the Minkowski connectivity increases very sharply until the maximum positive value at 2.0 (3.8) nm and then vanishes at 2.9 (4.3) nm. The Minkowski connectivity of the image of the healthy carotid tissue with G0 decreases more smoothly at *τ*_*χ*_ = 4.3 nm to the minimum negative value at *τ*_*χ*_ = 7.3 nm and then rises until the maximum positive value at 9.6 nm. After that, it decreases gradually until it vanishes at 15 nm. In contrast, the Minkowski connectivity of the image of the plain healthy carotid tissue shows fluctuations, with the minimum negative value at 21.2 nm and the maximum positive value at 30.6 nm. This is more pronounced for the Minkowski connectivity of the atheromatous carotid tissue, where several minima and maxima are demonstrated.

In Figure 14b (2 × 2 μm), the Minkowski connectivity of the plain atheromatous carotid tissue starts to decrease early at 7.5 nm to the minimum value at 52.5 nm and then rises to the maximum value at 86.8 nm, while the Minkowski connectivity of the plain asymptomatic carotid tissue displays a plateau of approximately 26 nm when it reaches the minimum and maximum values at 46 nm and 85 nm, respectively. Furthermore, the addition of G0 on the healthy tissue results in sharp peaks of the Minkowski connectivity, with a minimum negative value at 38.3 nm and maximum positive value at 42.8 nm. However, the addition of G0 on the atheromatous carotid tissue results in strong fluctuations of the Minkowski connectivity from approximately 136 nm to 205 nm when it starts vanishing. This phenomenon is remarkably reduced when conjugated dendrimers with ZnPc are placed on the atheromatous carotid tissue. In Figure 14c (3 × 3 μm), the Minkowski connectivity of the plain atheromatous carotid tissue has a minimum value at 78.7 nm and maximum value at 116.1 nm, while the Minkowski connectivity of the atheromatous carotid tissue with the added conjugated dendrimers with ZnPc displays a plateau of 45 nm at approximately 223 nm for negative values and another plateau of 45 nm at approximately 308 nm for maximum values.

In Figure 14d (5 × 5 μm), the Minkowski connectivity decreases very sharply at *τ*_*χ*_ = 16.6 nm to the minimum negative value at *τ*_*χ*_ = 18.8 nm for the images of G0 on glass with a concentration of 0.025 M. Then, the Minkowski connectivity increases very sharply until the maximum positive value at 19.4 nm and then vanishes approximately at 26 nm. Atheromatous carotid tissue with G0 and atheromatous carotid tissue with G0 conjugated with ZnPc show almost the same behaviour regarding the Minkowski connectivity, but the latter has fewer fluctuations. Concerning the plain asymptomatic carotid tissue, the Minkowski connectivity displays a broad plateau with several local minima for negative values at approximately 120 nm and two local maxima at 184 nm and 247 nm. In contrast, the Minkowski connectivity of the asymptomatic carotid tissue has a minimum value at 90 nm and then fluctuates around zero until it reaches a positive plateau from 155 nm to 195 nm. Finally, it gradually decreases to a zero value.

## Discussion

Normal vascular physiology results in tight (<2 nm) endothelial junctions, which will restrict nanoparticle distribution, whereas a dysfunctional endothelium leads to large gaps that allow NPs to penetrate the endothelial barrier, deposit at local sites and remain retained locally, owing to impaired lymphatic drainage [72]. This phenomenon, which occurs in pathological conditions such as cancer or atherosclerosis, is known as the enhanced permeation and retention effect (EPR) [12]. Nanoparticle size is one crucial determinant of accumulation and penetration into diseased tissue, and NPs with sub-100 nm sizes are optimal for the EPR [73]. Therefore, the adsorption and aggregation of G0 is a major parameter of nanodrug efficacy.

The adsorption and aggregation of dendrimers to mica and silica surfaces, particularly of higher generations, was studied by AFM in several works [74-78]. These studies revealed that such dendrimers flatten substantially upon adsorption and that sometimes aggregates can be observed on the surface, particularly at a higher pH. It was further reported that the PAMAM dendrimers adsorb on mica and silica in correlated liquid-like monolayers and that repulsive electrostatic interactions between the dendrimers lead to low adsorption densities.

Mecke et al. [74] showed experimentally using AFM and via atomistic molecular dynamics simulations that both charged and uncharged PAMAM dendrimers strongly adsorb to mica surfaces, resulting in deformation of the molecules. The heights of the molecules, as determined by AFM, were much less than the diameter in their spherically symmetric state and did not depend strongly on the charge of the branch ends. Flattening of the dendrimers also occurred at the liquid-solid interface, although to a lesser degree. Furthermore, they found that the PAMAM dendrimers in aqueous environments increase in height and volume due to swelling. Studies combining optical reflectometry and AFM demonstrated that the adsorption process of PAMAM dendrimers to silica substrates is driven by attractive forces between the dendrimers and the substrate [75]. Thus, the initial adsorption rate depended on the solution composition only weakly. However, the maximum adsorbed amount increased strongly with the ionic strength and pH.

In another AFM study of PAMAM dendrimers [76], it was noted that the drying procedure could induce the formation of aggregates by capillary forces and, therefore, influence the relative positions of the deposited dendrimers. Thus, under different conditions, the drying process could indeed result in a rearrangement within the adsorbed dendrimer layer. However, the authors noted that the amount of deposited dendrimers remained constant independent of the relative positions. Recently, Yu et al. [77] showed that aggregates’ morphology of the amphiphilic G1 PAMAM dendrimer onto the mica substrate was seemingly spherical at low concentration and that semi-continuous films or network structure films were formed at high concentrations. Finally, Müller et al. [78] stated that the dendrimer shapes depend on the substrate type and that the aqueous solvent may influence dendrimer conformation, as they observed the formation of large aggregates on a HOPG surface when water was used as a solvent using AFM.

Studies of PAMAM distribution on biological samples are rather scarce [79-82]. For example, Dobrovolskaia et al. [79] reported in *in vitro* studies the effects of the size and surface terminal groups of PAMAM dendrimers on healthy human platelets. The study revealed that only dendrimers of higher generations with positive charge, not negative or neutral or small cationic dendrimers, contributed to the aggregation of human platelets *in vitro*. It was stated that large cationic PAMAM dendrimers induce platelet aggregation through the disruption of membrane integrity. In another recent study of G4 PAMAM dendrimer-treated rat brains [80], AFM images showed evidence of dendrimers in the inner capillary wall and demonstrated that PAMAM dendrimers are present at the blood-brain barrier (BBB). Furthermore, it was found that, while the height of distinct NPs ranged only from 2.7 to 10 nm, the width was 7 to 13 times larger than the expected diameter of the G4 PAMAM dendrimers, indicating that the measured NPs could be aggregates of dendrimers. Furthermore, Kitchens et al. [81] found that the extravasation time of PAMAM-NH_2_ dendrimers (G1-G4) across the microvascular endothelium of the cremaster muscle of male golden Syrian hamsters (*Mesocricetusauratus*) is size dependent due to increased exclusion from the endothelial pores. Finally, Markowicz-Piasecka et al. [82], in the only study to the authors’ knowledge on the effect of PAMAM dendrimers on the endothelium of human arteries, found that the influence of PAMAM dendrimers on the process of clot formation and fibrinolysis depends on the concentration and generation of the dendrimer and that G3 and G4 PAMAM dendrimers adversely affected human endothelium.

Here, the AFM study of G0 and G0/ZnPc conjugates, followed by textural analysis in terms of the root mean square surface roughness (*R*_*q*_), the surface roughness (*R*_*a*_), the mean $\left(\overline{Z}\right),$ media (*Z*_1/2_), mode (*Z*_*mp*_) and range (*R*_*t*_) heights, the maximum valley depth (*R*_*mvd*_), the maximum peak height (*R*_*mph*_), skewness (*R*_*sk*_), kurtosis (*R*_*ku*_), fractal dimension and Minkowski functionals, shows interesting results. First, the adsorption and aggregation of G0 on human tissue differs greatly from those on a flat inorganic material. Indeed, from the AFM images and the corresponding statistical analysis, it is shown that G0 are more or less monodisperse on the latter, while they spread more uniformly on the human tissue. Furthermore, differences on the local morphological characteristics of plain symptomatic and asymptomatic tissues were found. These differences, in correlation with the EPR effect, lead to selective aggregation of the dendrimers to the atheromatous carotid tissues. This is evident from the opposite signs of the relative difference of the fractal dimensions as well as from the behaviour of the Minkowski functional curves.

The conjugation of ZnPcs to G0 provides significant changes of surface roughness parameters compared to the dendrimers alone and the skewness and kurtosis parameters show significant distortions of PDF’s and aggregation features under G0/ZnPc loading. It seems that the addition of the hydrophobic ZnPcs counterbalances the effects of PAMAM dendrimers on the atheromatous carotid tissue, suggesting that both the surface type and the drug composition mediate the drug delivery efficiency, in full agreement with previous nanothermodynamic studies of nanoparticles on surfaces [28,83].

**Figure 15:**
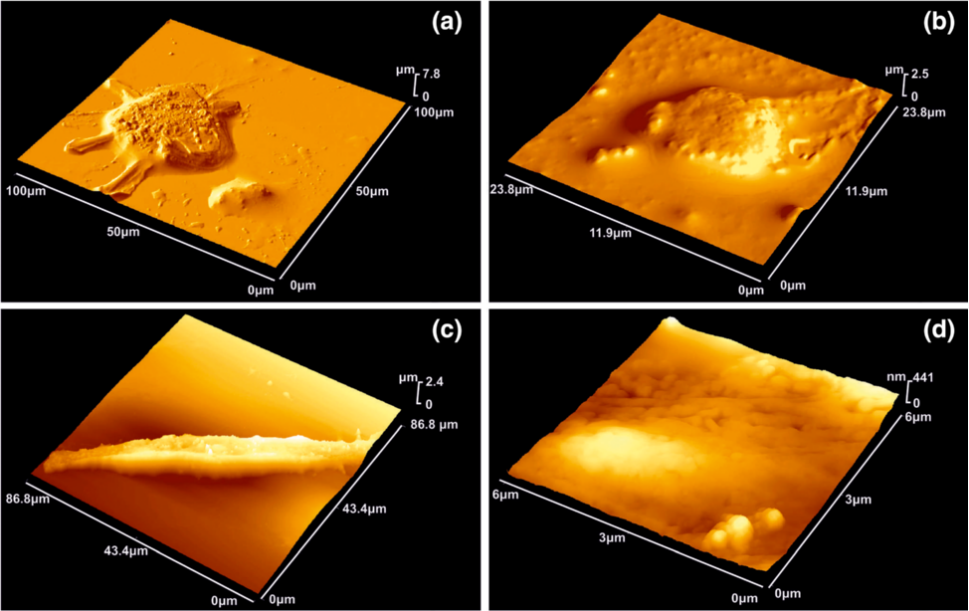
AFM images of individual endothelial, THP-1 and SMC cells. **(a)** Human Brachiocephalic Artery Endothelial Cell (HBcAEC-ECACC™) isolated from normal brachiocephalic arteries. **(b)** THP-1 (ATCC®TIB-202™) cell derived from a 1-year-old boy with leukemia. **(c)** Human Brachiocephalic Artery SMC cell (HBcASMC ECACC™) isolated from normal brachiocephalic arteries. **(d)** AFM image of endothelial cell at higher magnification. The statistical surface parameters of individual cells and healthy arteries differ significantly, Additional file 3, suggesting dissimilar nanodrug uptake efficiency between tissue and individual cell.

Indeed, it is known that phospholipids of the cellular membrane extend their hydrophilic head to the outer cell environment [12] and that the environment around a cell membrane is hydrophilic. The cell membrane is being spanned by transmembrane proteins; these proteins usually have their N-terminal domain extracellular, and the C-terminal domain is found in the inner cell. Thus, the application of the hydrophobic environment around a cell membrane could change the hydrophilic interactions between transmembrane proteins and the extracellular matrix (ECM), which leads to a partial disorder of balance in the cell and its outer environment due to different thermodynamic environments and polar-entropic competition at the nanoscale [83]. Nanoparticle encapsulation and change of local thermodynamic environment enhance image contrast in computer tomography of atheromatous plaque [84]. Finally, large morphological differences between tissue and individual endothelial, SMC and THP1 cells, Figure 15 and Additional file 3: Table S3.1, imply different nanodrug uptake efficiency by individual cells and further studies are needed towards this direction.

## Conclusions

In conclusion, the results of this work show that the addition of G0 PAMAM dendrimers or G0 PAMAM/ZnPc conjugates to the carotid tissue, either healthy or atheromatous, significantly alters the local texture characteristics of human carotids at the nanoscale, indicating different agglomerating responses of the G0 NPs. Most importantly, all statistical quantities showed that the deposition of nanodrug carriers on the healthy tissue seems to have an inverse impact in comparison to the deposition on atheromatous tissue, clearly suggesting the small size agglomeration of G0 NPs on the healthy tissue, with a particle size less than the surface roughness parameters of the tissue. In addition, different size agglomeration between G0 and G0/ZnPc and selective aggregation of the dendrimers to the atheromatous carotid tissues is observed. However, in addition to the size and composition of G0 aggregates, the penetration of dendrimers across the endothelial barrier depends on other important features, such as concentration, incubation time, efflux transport, surface charge, modification [85], local thermodynamic conditions [28] and polar-entropic competition [83]; therefore, future studies comprising all these parameters are necessary.

## Additional files

Additional file 1:
**Surface roughness parameters of healthy and atheromatous human tissues loaded with G0 and G0/ZnPc conjugated nanodrugs.** The root mean square surface roughness (*R*_*q*_), the surface roughness (*R*_*a*_), the mean $\left(\overline{\mathrm{Z}}\right),$ media (*Z*_1/2_), mode (*Z*_*mp*_) and range (*R*_*t*_) heights, the maximum valley depth (*R*_*mvd*_), the maximum peak height (*R*_*mph*_), skewness (*R*_*sk*_) and kurtosis (*R*_*ku*_) of healthy and atheromatous human tissues loaded with G0 PAMAM and G0/ZnPc conjugated nanodrugs are tabulated. Statistical analysis suggests that both the surface type and the drug composition mediate the drug delivery efficiency. Additional file 2:
**AFM images, line analysis and size distribution of ZnPc nanoparticles on Au and Si surfaces.** Size statistics of aggregation of ZnPc nanoparticles on gold (Au) and Si standard test surfaces is different than the aggregation of G0 on the same surfaces. ZnPc nanoparticles tend to aggregate at average sizes much higher than the surface roughness parameters of the test surfaces. This response is owing to the presence of electron charges on the surface of ZnPc nanoparticles and demonstrated by conductive atomic force microscopy. In case of conjugation of ZnPc with the G0 dendrimers, the negative charges are compensated to give nearly neutral nanoparticles of smaller size. Additional file 3:
**Surface roughness parameters of individual endothelial, THP-1 cells and SMC and preparation of cell cultures.** **Table S3.1.** Surface roughness parameters of individual endothelial, THP-1 cells and SMC. Surface roughness parameters of THP-1 (ATCC®TIB-202™), HBcAEC (ECACC) and HBcASMC (ECACC) cell lines. They are different from the atheromatous surface roughness parameters, indicating dissimilar nanodrug uptaking efficiency. **Table S3.2.** Preparation of cell cultures. THP-1 (ATCC®TIB-202™), HBcAEC (ECACC) and HBcASMC (ECACC) cell lines were used. All cell cultures were performed in an incubator at a 95% *w*/*w* ambient air, 5% *w*/*w* CO_2_ gaseous environment and the temperature is maintained at 37°C.
