# Supplementary material for: Selective aggregation of PAMAM dendrimer nanocarriers and PAMAM/ZnPc nanodrugs on human atheromatous carotid tissues: a photodynamic therapy for atherosclerosis
Source: Nanoscale Res Lett. 2015 May 7;10:210. doi: 10.1186/s11671-015-0904-5 (PMC4431993; doi:10.1186/s11671-015-0904-5)
Supplement: Additional file 1: — Surface roughness parameters of healthy and atheromatous human tissues loaded with G0 and G0/ZnPc conjugated nanodrugs. The root mean square surface roughness (R q), the surface roughness (R a), the mean $\left(\overline{\mathrm{Z}}\right),$Z¯, media (Z 1/2), mode (Z mp) and range (R t) heights, the maximum valley depth (R mvd), the maximum peak height (R mph), skewness (R sk) and kurtosis (R ku) of healthy and atheromatous human tissues loaded with G0 PAMAM and G0/ZnPc conjugated nanodrugs are tabulated. Statistical analysis suggests that both the surface type and the drug composition mediate the drug delivery efficiency. [file 11671_2015_904_MOESM1_ESM.docx]

**Additional file 1**

**Surface roughness parameters of healthy and atheromatous human tissues loaded with G0 and G0/ZnPc conjugated nanodrugs.**

The root mean square surface roughness ($R_{q})$, the surface roughness${(R}_{a})$, the mean $\left( \bar{Z} \right),$ media $\left( Z_{1/2} \right),$ mode ${(Z}_{mp})$ and range ${(R}_{t}$) heights, the maximum valley depth ${(R}_{mvd}$), the maximum peak height ${(R}_{mph})$, skewness ${(R}_{sk})$ and kurtosis ${(R}_{ku})$ of healthy and atheromatous human tissues loaded with G0 PAMAM and G0/ZnPc conjugated nanodrugs are tabulated. Statistical analysis suggests that both the surface type and the drug composition mediate the drug delivery efficiency.

**Table 1.1** Surface characteristics obtained from AFM images of 1μm x 1μm samples

| Size | | **1 µm x 1 µm** | | | | |
| --- | --- | --- | --- | --- | --- | --- |
| Number of points  N (pixels) | | 512 x 512 | | | | |
| Description | | Healthy | Atheromatous | Healthy + PAMAM | Glass +  PAMAM  (0.05M) | Glass +  PAMAM  (0.025M) |
| SURFACE PARAMETERS | MEAN HEIGHT  $\bar{Z}=\frac{1}{N}\sum_{i=0}^{N} Z_{i}$ (nm) | **24.91** | **49.37** | **8.74** | **3.54** | **1.89** |
|  | RANGE  $R_{t}=Z_{max}-Z_{min}$ (nm) | **58.85** | **86.90** | **22.03** | **8.13** | **6.60** |
|  | MEDIAN HEIGHT $Z_{1/2}$  $\int_{-\infty}^{Z_{1/2}} f\left( Z \right)dZ=\int_{Z_{1/2}}^{\infty} f\left( Z \right)dZ=1/2$ (nm) | **24.63** | **48.51** | **8.62** | **3.51** | **1.87** |
|  | MODE HEIGHT $Z_{mp}$  $\left[ \frac{d}{dZ}\int_{-\infty}^{\infty} f\left( Z \right)dZ \right]_{Z=Z_{mp}}=0$ (nm) | **23.77** | **45.94** | **8.40** | **3.50** | **1.86** |
|  | RMS ROUGHNESS  $R_{q}=\sqrt{\frac{1}{N}\sum_{i=0}^{N} \left\vert Z_{i}-\bar{Z} \right\vert^{2}}$ (nm) | **9.96** | **10.12** | **2.18** | **0.79** | **0.38** |
|  | AVERAGE ROUGHNESS  $R_{a}=\frac{1}{N}\sum_{i=0}^{N} \left\vert Z_{i}-\bar{Z} \right\vert$ (nm) | **7.78** | **7.87** | **1.62** | **0.53** | **0.20** |
|  | MAX VALLEY DEPTH  $R_{mvd}=\left\vert{{(Z}_{i}-\bar{Z})}_{min} \right\vert$ (nm) | **24.91** | **49.37** | **8.74** | **3.54** | **1.89** |
|  | MAX PEAK HEIGHT  $R_{mph}{{=(Z}_{i}-\bar{Z})}_{max}$ (nm) | **34.05** | **37.53** | **13.29** | **4.59** | **4.71** |
|  | SKEWNESS  $R_{sk}=\frac{1}{NR_{q}^{3}}\sum_{i=0}^{N} \left( Z_{i}-\bar{Z} \right)^{3}$ | **0.16** | **0.09** | **0.57** | **0.79** | **4.94** |
|  | KURTOSIS  $R_{ku}=\frac{1}{NR_{q}^{4}}\sum_{i=0}^{N} \left( Z_{i}-\bar{Z} \right)^{4}$ | **3.00** | **3.55** | **5.38** | **7.51** | **46.20** |

**Table 1.2** Surface characteristics obtained from AFM images of 2μm x 2μm samples

| Size | | **2 µm x 2 µm** | | | | |
| --- | --- | --- | --- | --- | --- | --- |
| Number of points  N (pixels) | | 512 x 512 | | | | |
| Description | | Healthy | Atheromatous | Healthy + PAMAM | Atheromatous  + PAMAM | Atheromatous  + PAMAM  +ZnPc |
| SURFACE PARAMETERS | MEAN HEIGHT  $\bar{Z}=\frac{1}{N}\sum_{i=0}^{N} Z_{i}$ (nm) | **78.92** | **56.46** | **41.49** | **166.63** | **175.17** |
|  | RANGE  $R_{t}=Z_{max}-Z_{min}$ (nm) | **172.12** | **119.47** | **66.71** | **248.38** | **326.79** |
|  | MEDIAN HEIGHT $Z_{1/2}$  $\int_{-\infty}^{Z_{1/2}} f\left( Z \right)dZ=\int_{Z_{1/2}}^{\infty} f\left( Z \right)dZ=1/2$ (nm) | **79.10** | **55.70** | **41.42** | **166.52** | **176.75** |
|  | MODE HEIGHT $Z_{mp}$  $\left[ \frac{d}{dZ}\int_{-\infty}^{\infty} f\left( Z \right)dZ \right]_{Z=Z_{mp}}=0$ (nm) | **87.27** | **45.95** | **42.42** | **156.81** | **192.04** |
|  | RMS ROUGHNESS  $R_{q}=\sqrt{\frac{1}{N}\sum_{i=0}^{N} \left\vert Z_{i}-\bar{Z} \right\vert^{2}}$ (nm) | **20.12** | **16.41** | **5.48** | **24.09** | **41.97** |
|  | AVERAGE ROUGHNESS  $R_{a}=\frac{1}{N}\sum_{i=0}^{N} \left\vert Z_{i}-\bar{Z} \right\vert$ (nm) | **15.77** | **12.90** | **3.65** | **17.23** | **32.37** |
|  | MAX VALLEY DEPTH  $R_{mvd}=\left\vert{{(Z}_{i}-\bar{Z})}_{min} \right\vert$ (nm) | **78.92** | **56.46** | **41.49** | **166.63** | **175.17** |
|  | MAX PEAK HEIGHT  $R_{mph}{{=(Z}_{i}-\bar{Z})}_{max}$ (nm) | **93.20** | **63.01** | **25.22** | **81.75** | **151.62** |
|  | SKEWNESS  $R_{sk}=\frac{1}{NR_{q}^{3}}\sum_{i=0}^{N} \left( Z_{i}-\bar{Z} \right)^{3}$ | **0.00** | **0.26** | **0.04** | **-1.50** | **-0.03** |
|  | KURTOSIS  $R_{ku}=\frac{1}{NR_{q}^{4}}\sum_{i=0}^{N} \left( Z_{i}-\bar{Z} \right)^{4}$ | **3.43** | **3.31** | **7.75** | **9.12** | **3.57** |

**Table 1.3** Surface characteristics obtained from AFM images of 3 μm x 3 μm samples

| Size | | **3 µm x 3 µm** | |
| --- | --- | --- | --- |
| Number of points  N (pixels) | | 512 x 512 | |
| Description | | Atheromatous | Atheromatous +  PAMAM + ZnPc |
| SURFACE PARAMETERS | MEAN HEIGHT  $\bar{Z}=\frac{1}{N}\sum_{i=0}^{N} Z_{i}$ (nm) | **102.26** | **280.84** |
|  | RANGE  $R_{t}=Z_{max}-Z_{min}$ (nm) | **177.38** | **477.28** |
|  | MEDIAN HEIGHT $Z_{1/2}$  $\int_{-\infty}^{Z_{1/2}} f\left( Z \right)dZ=\int_{Z_{1/2}}^{\infty} f\left( Z \right)dZ=1/2$ (nm) | **100.96** | **281.19** |
|  | MODE HEIGHT $Z_{mp}$  $\left[ \frac{d}{dZ}\int_{-\infty}^{\infty} f\left( Z \right)dZ \right]_{Z=Z_{mp}}=0$ (nm) | **92.02** | **279.77** |
|  | RMS ROUGHNESS  $R_{q}=\sqrt{\frac{1}{N}\sum_{i=0}^{N} \left\vert Z_{i}-\bar{Z} \right\vert^{2}}$ (nm) | **21.22** | **60.30** |
|  | AVERAGE ROUGHNESS  $R_{a}=\frac{1}{N}\sum_{i=0}^{N} \left\vert Z_{i}-\bar{Z} \right\vert$ (nm) | **16.96** | **46.62** |
|  | MAX VALLEY DEPTH  $R_{mvd}=\left\vert{{(Z}_{i}-\bar{Z})}_{min} \right\vert$ (nm) | **102.26** | **280.84** |
|  | MAX PEAK HEIGHT  $R_{mph}{{=(Z}_{i}-\bar{Z})}_{max}$ (nm) | **75.12** | **196.44** |
|  | SKEWNESS  $R_{sk}=\frac{1}{NR_{q}^{3}}\sum_{i=0}^{N} \left( Z_{i}-\bar{Z} \right)^{3}$ | **0.18** | **-0.17** |
|  | KURTOSIS  $R_{ku}=\frac{1}{NR_{q}^{4}}\sum_{i=0}^{N} \left( Z_{i}-\bar{Z} \right)^{4}$ | **3.23** | **4.00** |

**Table 1.4** Surface characteristics obtained from AFM images of 5 μm x 5 μm samples

| Size | | **5 µm x 5 µm** | | | | |
| --- | --- | --- | --- | --- | --- | --- |
| Number of points  N (pixels) | | 512 x 512 | | | | |
| Description | | Healthy | Atheromatous | Atheromatous + PAMAM | Atheromatous + PAMAM + ZnPc | Glass +  PAMAM  (0.025M) |
| SURFACE PARAMETERS | MEAN HEIGHT  $\bar{Z}=\frac{1}{N}\sum_{i=0}^{N} Z_{i}$ (nm) | **133.98** | **166.30** | **266.32** | **260.82** | **19.23** |
|  | RANGE  $R_{t}=Z_{max}-Z_{min}$ (nm) | **253.66** | **350.90** | **523.15** | **557.26** | **67.47** |
|  | MEDIAN HEIGHT $Z_{1/2}$  $\int_{-\infty}^{Z_{1/2}} f\left( Z \right)dZ=\int_{Z_{1/2}}^{\infty} f\left( Z \right)dZ=1/2$ (nm) | **137.79** | **167.05** | **266.98** | **255.94** | **19.02** |
|  | MODE HEIGHT $Z_{mp}$  $\left[ \frac{d}{dZ}\int_{-\infty}^{\infty} f\left( Z \right)dZ \right]_{Z=Z_{mp}}=0$ (nm) | **141.87** | **167.79** | **248.45** | **246.29** | **19.13** |
|  | RMS ROUGHNESS  $R_{q}=\sqrt{\frac{1}{N}\sum_{i=0}^{N} \left\vert Z_{i}-\bar{Z} \right\vert^{2}}$ (nm) | **43.20** | **39.06** | **65.86** | **87.03** | **6.57** |
|  | AVERAGE ROUGHNESS  $R_{a}=\frac{1}{N}\sum_{i=0}^{N} \left\vert Z_{i}-\bar{Z} \right\vert$ (nm) | **34.58** | **30.81** | **50.96** | **69.35** | **2.98** |
|  | MAX VALLEY DEPTH  $R_{mvd}=\left\vert{{(Z}_{i}-\bar{Z})}_{min} \right\vert$ (nm) | **133.98** | **166.30** | **266.32** | **260.82** | **19.23** |
|  | MAX PEAK HEIGHT  $R_{mph}{{=(Z}_{i}-\bar{Z})}_{max}$ (nm) | **119.68** | **184.60** | **256.83** | **296.44** | **48.23** |
|  | SKEWNESS  $R_{sk}=\frac{1}{NR_{q}^{3}}\sum_{i=0}^{N} \left( Z_{i}-\bar{Z} \right)^{3}$ | **-0.33** | **0.05** | **0.01** | **0.27** | **3.15** |
|  | KURTOSIS  $R_{ku}=\frac{1}{NR_{q}^{4}}\sum_{i=0}^{N} \left( Z_{i}-\bar{Z} \right)^{4}$ | **2.90** | **3.16** | **3.55** | **3.05** | **20.06** |
