# Supplementary material for: Selective aggregation of PAMAM dendrimer nanocarriers and PAMAM/ZnPc nanodrugs on human atheromatous carotid tissues: a photodynamic therapy for atherosclerosis
Source: Nanoscale Res Lett. 2015 May 7;10:210. doi: 10.1186/s11671-015-0904-5 (PMC4431993; doi:10.1186/s11671-015-0904-5)
Supplement: Additional file 2: — AFM images, line analysis and size distribution of ZnPc nanoparticles on Au and Si surfaces. Size statistics of aggregation of ZnPc nanoparticles on gold (Au) and Si standard test surfaces is different than the aggregation of G0 on the same surfaces. ZnPc nanoparticles tend to aggregate at average sizes much higher than the surface roughness parameters of the test surfaces. This response is owing to the presence of electron charges on the surface of ZnPc nanoparticles and demonstrated by conductive atomic force microscopy. In case of conjugation of ZnPc with the G0 dendrimers, the negative charges are compensated to give nearly neutral nanoparticles of smaller size. [file 11671_2015_904_MOESM2_ESM.docx]

**Additional file 2**

**AFM images, line analysis and size distribution of ZnPc nanoparticles on Au and Si** **surfaces.**

Size statistics of aggregation of ZnPc nanoparticles on gold (Au) and Si standard test surfaces is different than the aggregation of G0 on the same surfaces. ZnPc nanoparticles tend to aggregate at average sizes much higher than the surface roughness parameters of the test surfaces. This response is owing to the presence of electron charges on the surface of ZnPc nanoparticles and demonstrated by conductive atomic force microscopy. In case of conjugation of ZnPc with the G0 dendrimers, the negative charges are compensated to give nearly neutral nanoparticles of smaller size.

**2.1 Size analysis of ZnPc on Au surface.**

# ZnPc has poor solubility in PBS and consequently it aggregates at relatively larger agglomerations. The area consists of aggregations with different sizes, Figure 2.1-2.4. The aggregations are formed from particles with 70 *nm* average diameter and a mean z-height of 57 *nm*.

| 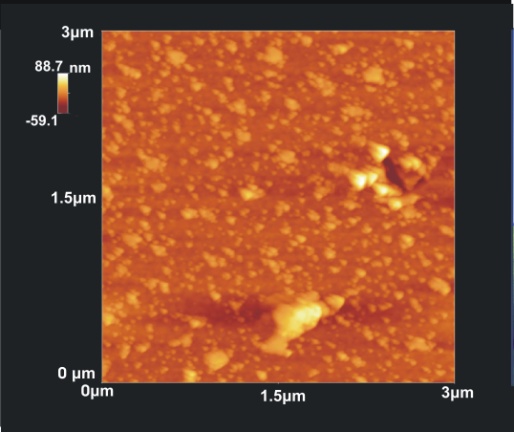  **Figure 2.1** AFM image (3 *μm* x 3 *μm*) of ZnPc aggregates on Au. |
| --- |

| 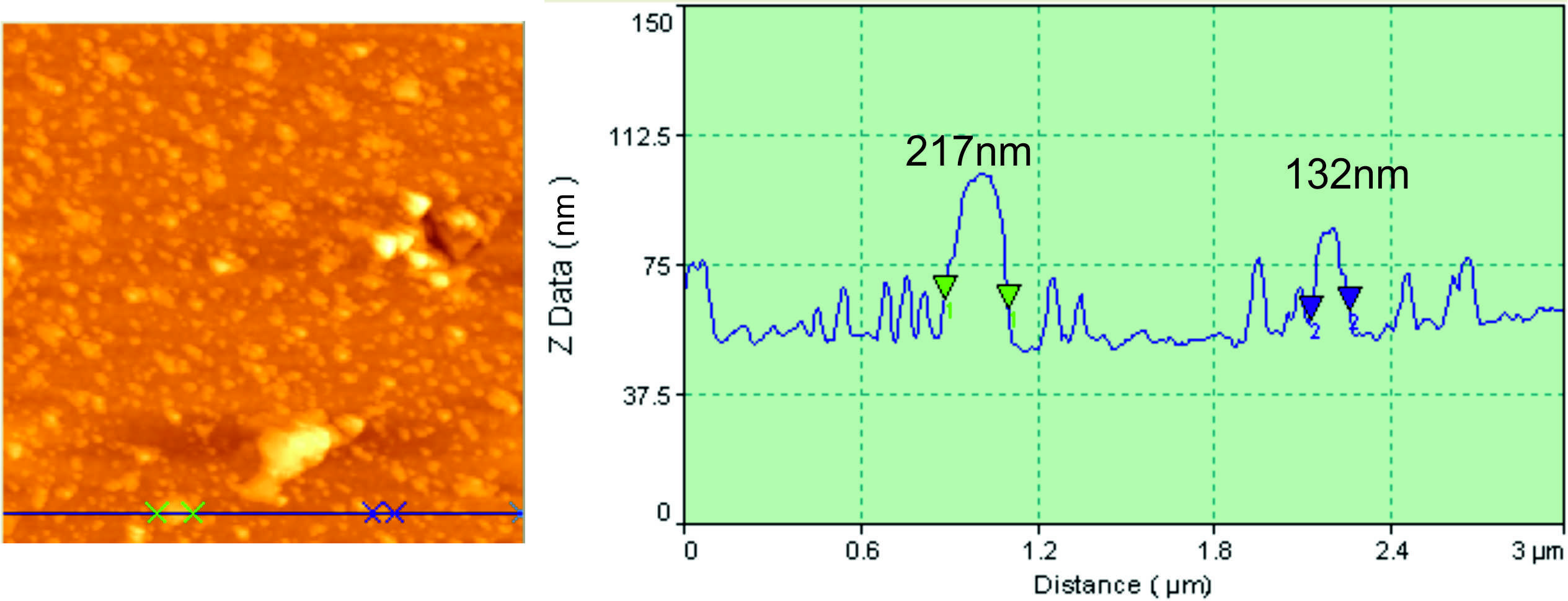 | 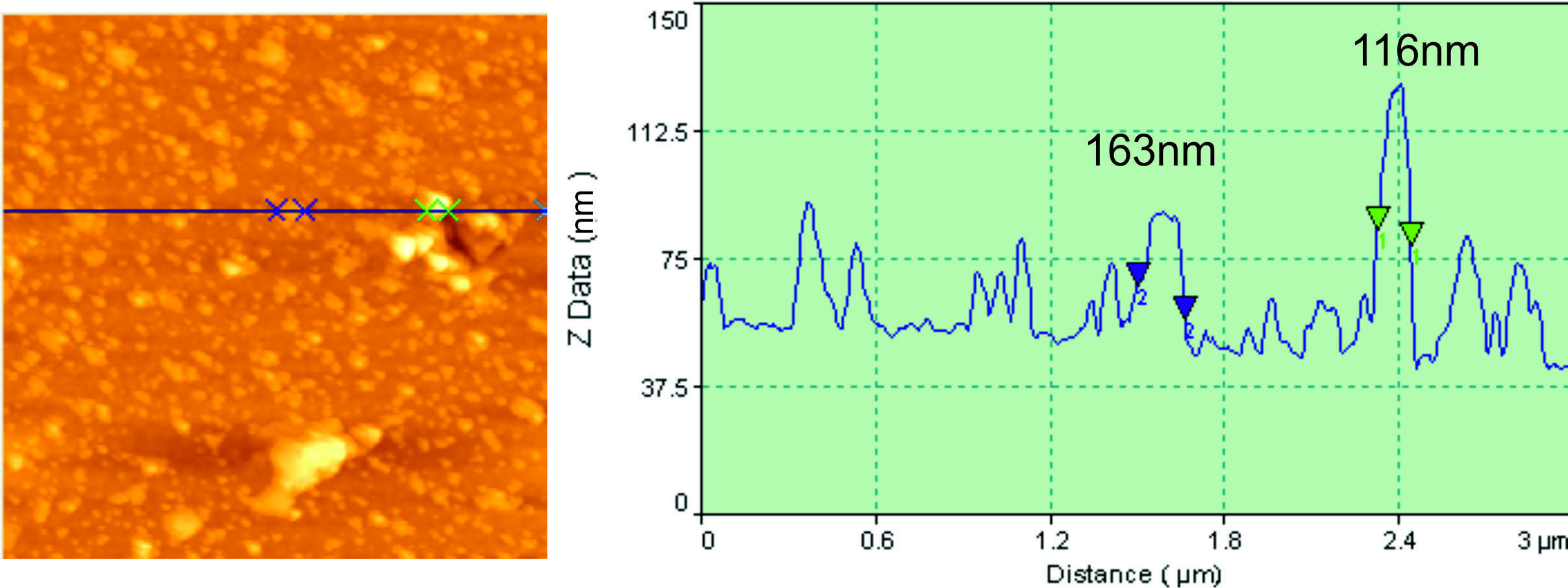 |
| --- | --- |
| 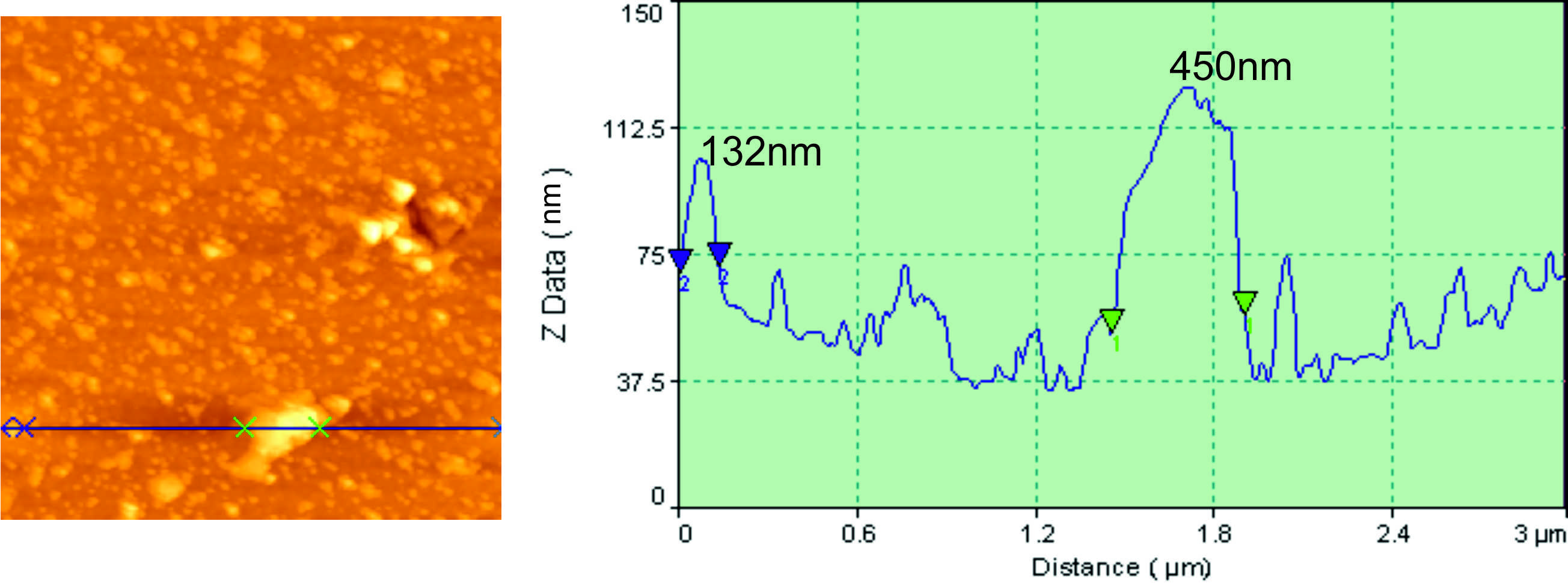 | 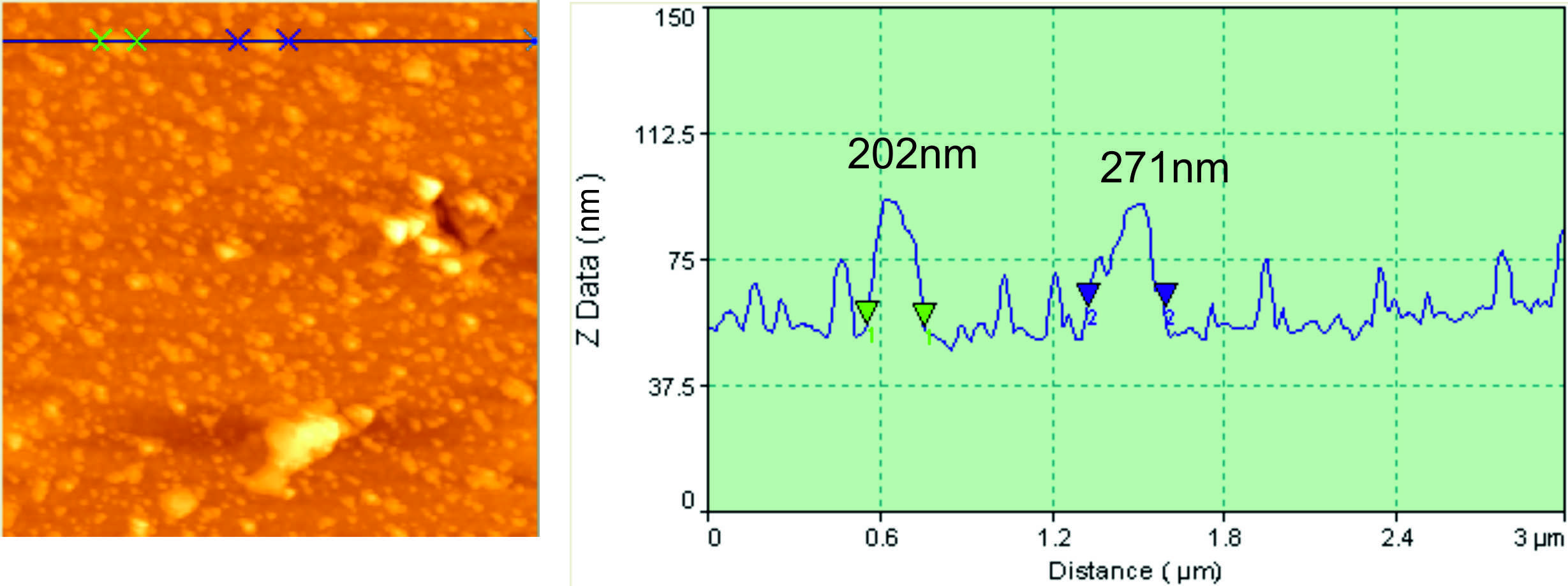 |
| **Figure 2.2** Line profile analysis of the AFM image of Figure 2.1 with different size aggregates. | |

| 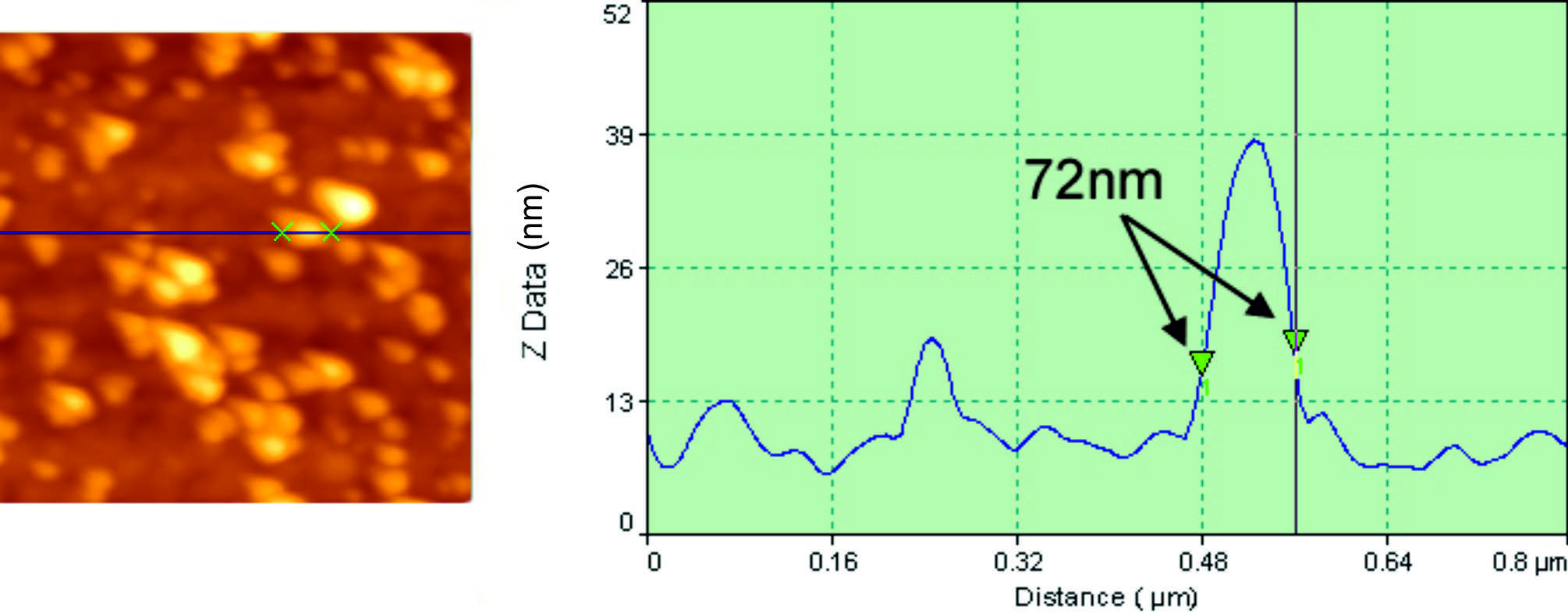  **Figure 2.3** Left: AFM image (0.8 *μm* x 0.8 *μm*) of ZnPc. Right: line profile analysis. | 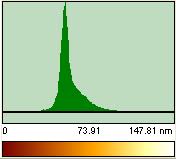  **Figure 2.4** Height distribution histogram of the ZnPc aggregates. |
| --- | --- |

**2.2 Size analysis of ZnPc on Si surface.**

| *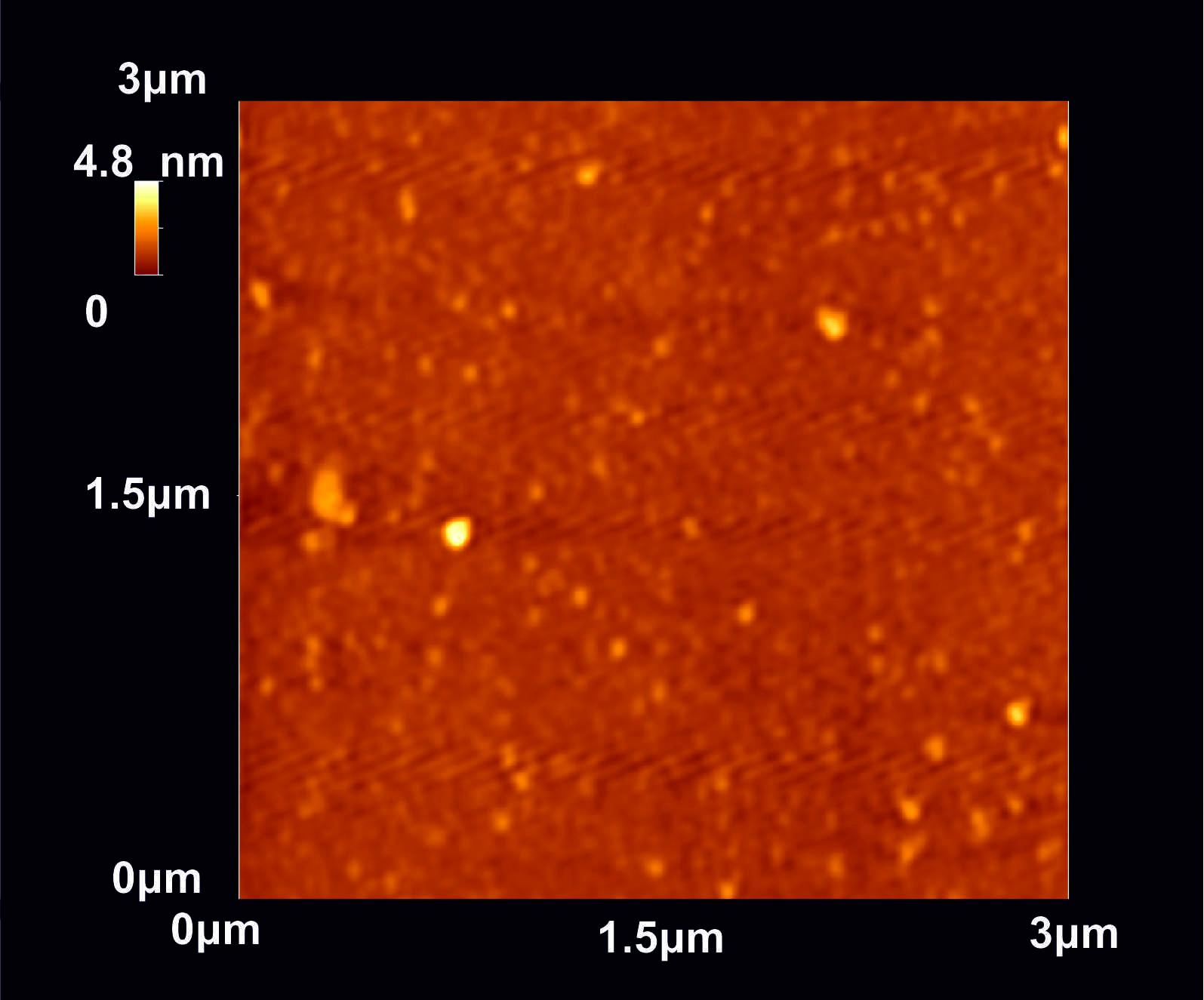* |
| --- |

**Figure 2.5** AFM image (3 *μm* x 3 *μm* ) of ZnPc aggregates on Si.


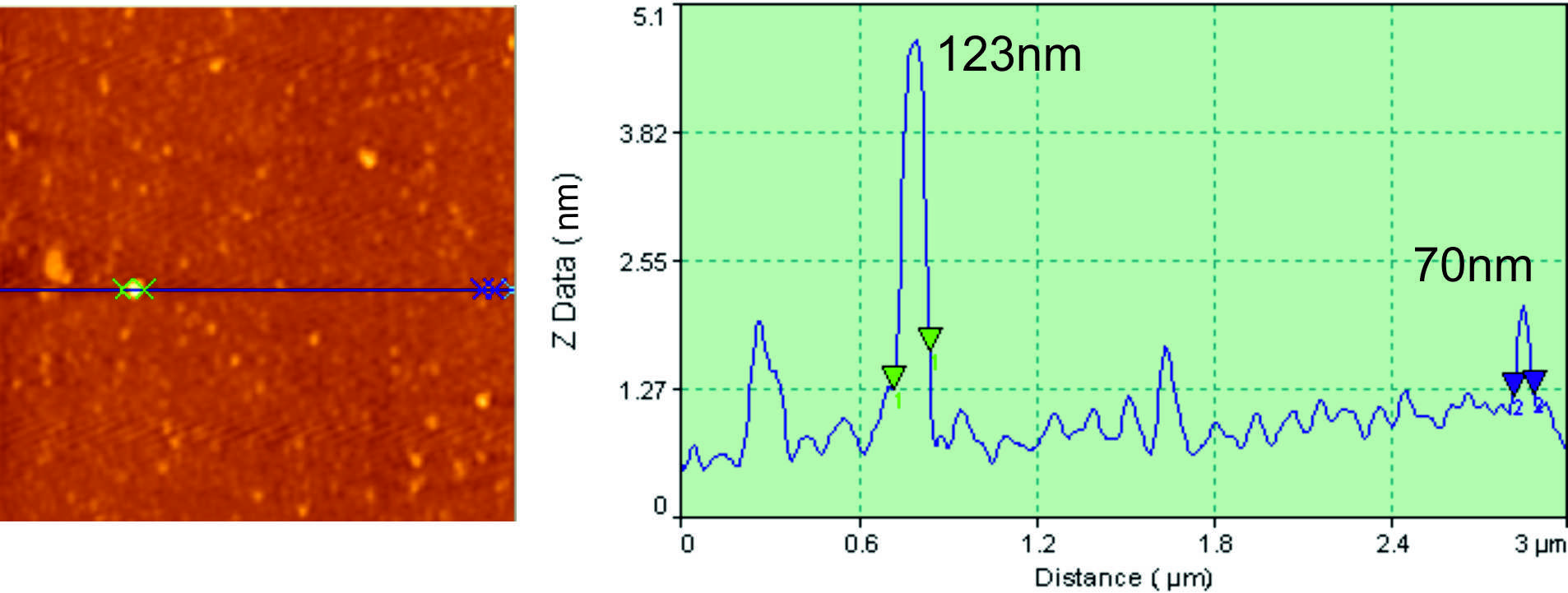

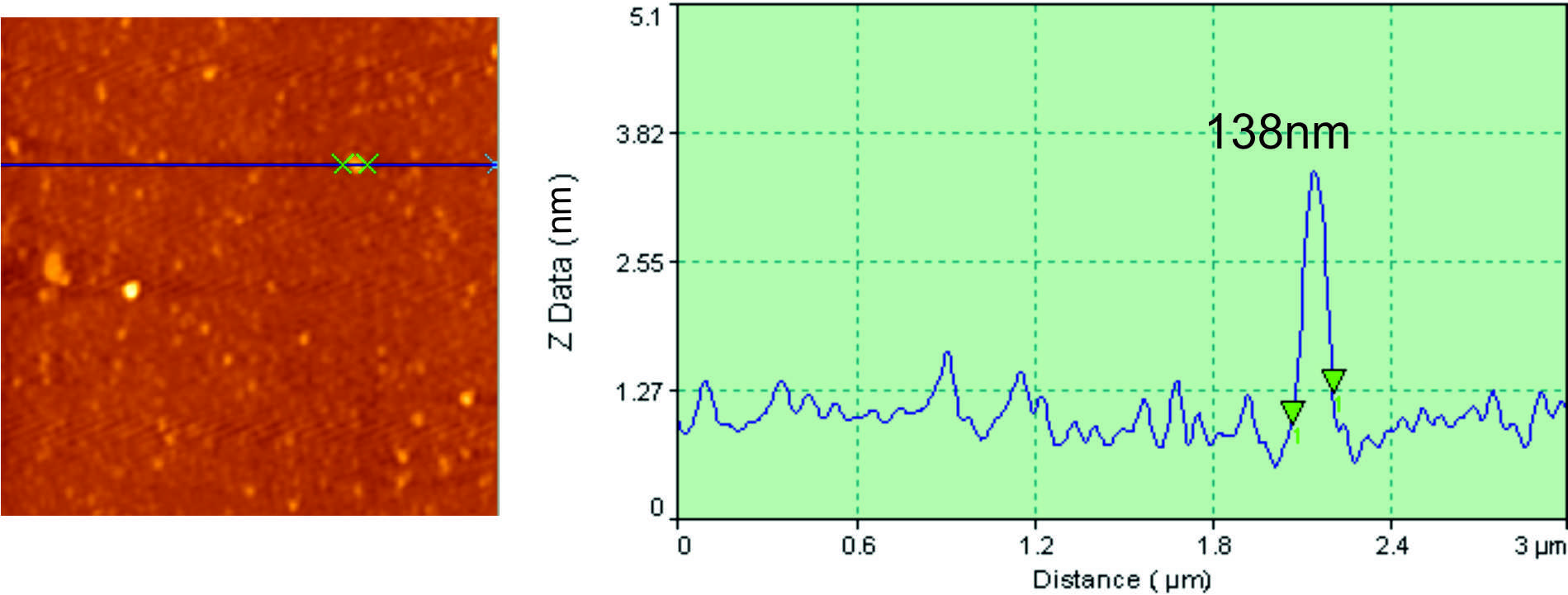

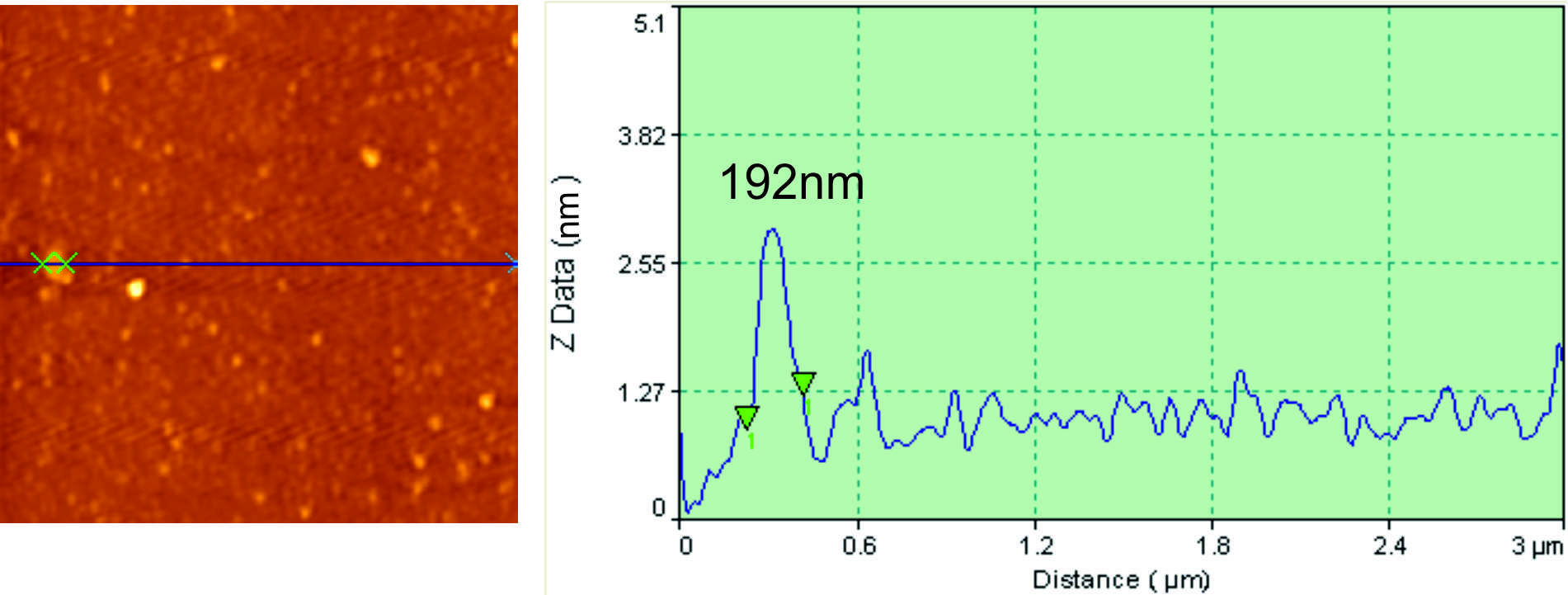


**Figure 2.6** Left: AFM image (3 *μm* x 3 *μm*) of ZnPc nanoaggregates on Si. Right: line profile analysis.

**Figure 2.7** Current-Voltage characteristic of a ZnPc aggregate on Au taken by conductive atomic force microscopy, with semiconductive response of nanodomains.
