# Supplementary material for: Selective aggregation of PAMAM dendrimer nanocarriers and PAMAM/ZnPc nanodrugs on human atheromatous carotid tissues: a photodynamic therapy for atherosclerosis
Source: Nanoscale Res Lett. 2015 May 7;10:210. doi: 10.1186/s11671-015-0904-5 (PMC4431993; doi:10.1186/s11671-015-0904-5)
Supplement: Additional file 3: — Surface roughness parameters of individual endothelial, THP-1 cells and SMC and preparation of cell cultures. Table S3.1. Surface roughness parameters of individual endothelial, THP-1 cells and SMC. Surface roughness parameters of THP-1 (ATCC®TIB-202™), HBcAEC (ECACC) and HBcASMC (ECACC) cell lines. They are different from the atheromatous surface roughness parameters, indicating dissimilar nanodrug uptaking efficiency. Table S3.2. Preparation of cell cultures. THP-1 (ATCC®TIB-202™), HBcAEC (ECACC) and HBcASMC (ECACC) cell lines were used. All cell cultures were performed in an incubator at a 95% w/w ambient air, 5% w/w CO2 gaseous environment and the temperature is maintained at 37°C. [file 11671_2015_904_MOESM3_ESM.docx]

**Additional file 3**

**3.1 Surface roughness parameters of individual endothelial, THP-1 cells and SMC.**

Surface roughness parameters of THP-1 (ATCC®TIB-202^™^), HBcAEC (ECACC), HBcASMC (ECACC) cell lines.

**Table 3.1** Surface characteristics obtained from parts of AFM images of individual cells

| Description | | Endothelial cell | THP-1 cell | SMC |
| --- | --- | --- | --- | --- |
| Sample Size | | **10 µm x 10 µm** | **5 µm x 5 µm** | **6 µm x 6 µm** |
| Number of points  N (pixels) | | 500 x 500 | | |
| SURFACE PARAMETERS | MEAN HEIGHT  $\bar{Z}=\frac{1}{N}\sum_{i=0}^{N} Z_{i}$ (nm) | **503.79** | **228.81** | **190.36** |
|  | RANGE  $R_{t}=Z_{max}-Z_{min}$ (nm) | **1005.20** | **364.50** | **440.75** |
|  | MEDIAN HEIGHT $Z_{1/2}$  $\int_{-\infty}^{Z_{1/2}} f\left( Z \right)dZ=\int_{Z_{1/2}}^{\infty} f\left( Z \right)dZ=1/2$ (nm) | **492.80** | **235.90** | **175.39** |
|  | MODE HEIGHT $Z_{mp}$  $\left[ \frac{d}{dZ}\int_{-\infty}^{\infty} f\left( Z \right)dZ \right]_{Z=Z_{mp}}=0$ (nm) | **450.20** | **234.70** | **137.20** |
|  | RMS ROUGHNESS  $R_{q}=\sqrt{\frac{1}{N}\sum_{i=0}^{N} \left\vert Z_{i}-\bar{Z} \right\vert^{2}}$ (nm) | **188.56** | **61.03** | **75.87** |
|  | AVERAGE ROUGHNESS  $R_{a}=\frac{1}{N}\sum_{i=0}^{N} \left\vert Z_{i}-\bar{Z} \right\vert$ (nm) | **152.60** | **46.36** | **62.05** |
|  | MAX VALLEY DEPTH  $R_{mvd}=\left\vert{{(Z}_{i}-\bar{Z})}_{min} \right\vert$ (nm) | **503.79** | **228.81** | **190.36** |
|  | MAX PEAK HEIGHT  $R_{mph}{{=(Z}_{i}-\bar{Z})}_{max}$ (nm) | **501.41** | **135.69** | **250.39** |
|  | SKEWNESS  $R_{sk}=\frac{1}{NR_{q}^{3}}\sum_{i=0}^{N} \left( Z_{i}-\bar{Z} \right)^{3}$ | **0.27** | **-0.51** | **0.54** |
|  | KURTOSIS  $R_{ku}=\frac{1}{NR_{q}^{4}}\sum_{i=0}^{N} \left( Z_{i}-\bar{Z} \right)^{4}$ | **2.55** | **3.12** | **2.57** |

**3.2 Preparation of cell cultures**

In this work, the THP-1 (ATCC®TIB-202^™^), HBcAEC (ECACC), HBcASMC (ECACC) cell lines were used. All cell cultures were performed in an incubator at a 95% w/w ambient air, 5 % w/w CO_2_ gaseous environment and the temperature is maintained at 37^o^ *C.*

THP-1 (ATCC^®^TIB-202^™^) cells are monocyte-like suspended cells and they were derived from a one year old boy with leukemia. The medium is RPMI 1640 serum with 2 *mM* L-glutamine adjusted to contain 1.5 *g*/*L* sodium bicarbonate, 4.5 *g*/*L* glucose and 10 *mM* HEPES. The cells are seeded on cover-slips coated with Poly-L-Lysine (PLL) 100 μg/ml in Phosphate buffer saline 10mM; pH 7.4(PBS) to make them adherent. The second cell line, Human Brachiocephalic Artery Endothelial Cells (HBcAEC-ECACC™) are primary endothelial cells isolated from normal brachiocephalic arteries and grown adhered. The medium is MesoEndo Cell growth medium (ECACC™) supplemented with FBS, growth factors, trace elements and antibiotics. The third cell line adherent cells, Human Brachiocephalic Artery SMC (HBcASMC ECACC™) are primary SMC isolated from normal brachiocephalic arteries and is also adhered. The medium is SMC Growth Medium (ECACC™) and is fully supplemented with amino acids, vitamins, minerals, organic compounds, inorganic salts, trace elements, fetal bovine serum, growth factors and antibiotics.
